# Near infrared light mediated photochemotherapy for efficiently treating deep orthotopic tumors guided by ultrasound imaging

**DOI:** 10.1080/10717544.2017.1375574

**Published:** 2017-09-25

**Authors:** Zuhua Wang, Shaoyan Xuan, Wenqi Qiu, Jiang Zhu, Xiaomeng Guo, Wei Li, Hanbo Zhang, Xiuliang Zhu, Yong-Zhong Du, Jian You

**Affiliations:** a College of Pharmaceutical Sciences, Zhejiang University, Hangzhou, People’s Republic of China;; b College of Pharmaceutical Sciences, Guiyang College of Traditional Chinese Medicine, Guiyang, People’s Republic of China;; c Department of Pharmacy, Shaoxing People’s Hospital, Shaoxing Hospital of Zhejiang University, Shaoxing, People’s Republic of China;; d The First Affiliated Hospital, Zhejiang University School of Medicine, Hangzhou, Zhejiang, People’s Republic of China;; e Sir Run Run Shaw Hospital, Zhejiang University School of Medicine, Hangzhou, People’s Republic of China;; f Department of Radiology, The Second Affiliated Hospital, Zhejiang University School of Medicine, Hangzhou, Zhejiang, People’s Republic of China

**Keywords:** Photothermal-chemotherapy, orthotopic tumor models, EphB4 expression, B-mode ultrasound imaging, hollow gold nanospheres

## Abstract

Recently, Combined cancer photothermal-chemotherapy has become a highly promising strategy in cancer treatment for its enhanced therapeutic efficacy, controlled drug release and reduced systemic toxicity. Almost all the reported strategies based on photothermal-chemotherapy have only focused on the treatment of superficial or subcutaneous cancer, which are not considered as a more clinically relevant and better predictive models of drug efficacy than orthotopic tumor models. Here, we reported an EphB4 receptor-targeting polymeric nanoplatform containing hollow gold nanospheres (HAuNS) and the anticancer drug paclitaxel (PTX) for cancer photothermal-chemotherapy. With the modification of the TNYL peptide, HP-TCS could specifically internalize into EphB4-positive SKOV3 and CT26 cells, further inducing the selective killing of the cells in co-cultured system, namely, EphB4-positive and EphB4-negative cells. Obvious targeting of the micelles into implanted orthotopic or subcutaneous tumors with high EphB4 expression was observed. Interestingly, increased accumulation of HP-TCS was observed in orthotopic colon tumors when compared with ectopic tumors. Highly specific accumulation of HP-TCS in EphB4-positive tumors significantly increased the feasibility of photothermal-chemotherapy mediated by the near infrared reflection (NIR) laser. Then, a systemic antitumor efficiency study was performed in implanted subcutaneous and visual orthotopic tumor models. Precise NIR laser irradiation could be localized on tumors under the guidance of B-mode ultrasound imaging, causing a rapid photothermal ablation effect limited to the region of tumors. Tumor growth was significantly inhibited by the photothermal-chemotherapy due to the triggered release of PTX. Our study provided a promising strategy of NIR laser-mediated photothermal-chemotherapy based on HP-TCS against the tumors (specially, deep orthotopic tumors) with high EphB4 expression.

## Introduction

Hyperthermia in the combined treatment of cancer has attracted great attention during the last two decades (Wust et al., 2002; Chen et al., 2016b; Moy & Tunnell, 2017). At present, chemotherapy is still the main method of cancer treatment, but chemotherapeutic agents typically lack selectivity, thus leading to a low efficacy of chemotherapy in cancer cells and high toxicity to normal cells (Greish, 2007). In addition, long-term repetitive chemotherapy could result in resistance to a variable degree (Gottesman, 2002). Photothermal therapy (PTT) presented a novel cancer treatment strategy that employs photo-absorbing agents to generate hyperthermia from optical energy, leading to the ‘burning’ of tumor cells (Li et al., 2012). Recently, photothermal therapy combined with cancer chemotherapy (Photothermal-chemotherapy) has become a highly promising strategy in cancer treatment given its ideal features, such as enhanced therapeutic efficacy, controlled drug release and reduced systemic toxicity (Chen et al., 2016a). It was noted that most studies of photothermal-chemotherapy in cancer were investigated only at the cellular level or by using conventional subcutaneous xenograft tumor models, because photothermal combined treatment typically involved in the irradiation of tumors with near-infrared (NIR) light (Chen et al., 2016b). In addition, this method is very convenient for the treatment of subcutaneous xenograft tumor models (Liao et al., 2015). However, orthotopic tumor models are considered more clinically relevant and better predictive models of drug efficacy than standard subcutaneous models (Talmadge et al., 2007). Because tumor cells are implanted directly into the organ of origin, these tumors reflect the original situation (such as microenvironment) much better than conventional subcutaneous xenograft tumor models (Botella et al., 2012; Imparato et al., 2015). Unfortunately, to date, the evaluation of photothermal-chemotherapy has rarely been performed using orthotopic tumor models. Photothermal combined treatment is challenging for deep orthotopic tumors because it is difficult to provide precise irradiation without directly observing the location of the tumors. Furthermore, the system offers greater risks for the treatment of orthotopic tumors because the tumor cells are closely conjoined with normal tissues of important organs. Increased accumulation of therapeutic agents in tumors but not in surrounding normal tissue and delivering precise irradiation to the tumors with an optimized condition (e.g. power and duration) inducing selective hyperthermia in the tumor region are critical for safe and efficient photothermal-chemotherapy of cancer. In this study, stearic acid-grafted chitosan (CSO-SA), a glycolipid-like polymer micelle, was synthesized by a coupling reaction between the amino groups of chitosan oligosaccharide (CSO) and carboxyl group of stearic acid (SA), as reported in our previous study (You et al., 2007a,b). This system exhibited excellent internalization into tumor cells and was employed to encapsulate hollow gold nanospheres (HAuNS) and the anticancer drug paclitaxel (PTX) for further photothermal-chemotherapy in cancer (You et al., 2013). HAuNS represent a novel class of biocompatible carriers with potential applications in photothermal cancer therapy. These carriers exhibit plasmon absorption in the near-infrared (NIR) region and a strong photothermal conducting property (Lu et al., 2009). To enhance the tumor targeting ability of CSO-SA micelles, the targeted peptide TNYLFSPNGPIARAW (designated as TNYL) was conjugated onto the surface of CSO-SA micelles that displayed high binding affinity to EphB4 (Xiong et al., 2011; You et al., 2007a,b). The EphB4 receptor is a leading member of the largest known family of receptor tyrosine kinases that controls various pathological processes, including tumor progression and angiogenesis (Pasquale, 2005; Stammes et al., 2017). Overexpression of EphB4 has also been observed in numerous tumor types (Kumar et al., 2006, 2009; Xia et al., 2006). Therefore, EphB4 is a promising target for tumor-targeting delivery in our system. For photothermal-combined cancer chemotherapy, orthotopic and subcutaneous xenograft clone tumor models with EphB4-positive expression were successfully generated. The specific binding of the final nanoparticles with EphB4-positive cancer cells and their targeted accumulation into subcutaneous xenograft and orthotopic tumors were investigated in detail. The efficacy of photothermal-chemotherapy against orthotopic and subcutaneous xenograft clone tumors was evaluated under the guidance of ultrasound imaging after the intravenous administration of our nanoparticles.

## Methods

### Materials

Chitosan oligosaccharide (CSO) with an approximate 19.0 kDa average molecular weight was obtained by the enzymatic degradation of 95% deacetylated chitosan (Mw = 45.0 kDa), which was supplied by Yuhuan Marine Biochemistry Co., Ltd. (Zhejiang, China). Stearic acid (SA) was obtained from Shanghai Chemical Reagent Co. Ltd. (Shanghai, China). Sodium citrate (>99%), cobalt chloride hexahydrate (99.99%), sodium borohydride (99%), and chloroauric acid trihydrate (American Chemical Society reagent grade) were purchased from Thermo Fisher Scientific (Waltham, MA) and were used as received. Octadecyl-3-mercaptopionate (OMP) was obtained from Chemical Industry Co. (Japan). PTX was gifted from Zhejiang Hisun Pharmaceutical Co, Ltd. (Taizhou Zhejiang, China). Di-tert-butyl dicarbonate ((Boc)_2 _O) was purchased from Shanghai Medpep Co., Ltd., China. 1-Ethyl-3-(3-dimethylaminopropyl) carbodiimide (EDC), 4-dimethylaminopyridine (DMAP), N,N-disuccinimidyl carbonate (DSC), and 3-(4,5-dimethylthiazol-2-yl)-2,5 -diphenyltetrazolium bromide (MTT) were purchased from Sigma-Aldrich (St Louis, MO). D-Luciferin (Sciencelight Catalog#001) was purchased from Sciencelight Biology Science & Technology Co., Ltd. (Shanghai, China). Indocyanine green (ICG) was purchased from Tokyo Chemical Industry, Japan. The EphB4 antibody (20883-1-AP) was purchased from Proteintech Group Inc. (Wuhan, China). DiR (DilC18(7)) was acquired from Life Technologies Biotechnology (USA). NH_2_-PEG_2000_-NH_2_ was purchased from Sigma-Aldrich Inc. (St Louis, MO). TNYL (sequence: TNYLFSPNGPIARAW) was provided from Baiaotai Biotechnology Inc. (Guangzhou, China). Trypsin was purchased from Gibco BRL, USA. Fetal bovine serum (FBS) was purchased from Sijiqing Biologic, China. All other solvents were of analytical or chromatographic grade.

### Preparation of HAuNS and PTX-loaded TNYL-CSO-SA micelles

OMP-HAuNS and PTX-loaded TNYL-CSO-SA micelles (HP-TCS) were prepared using dialysis methods (You et al., 2007a,b). Briefly, OMP-HAuNS (0.5 mL of 100 OD), PTX (2 mg) and TNYL-CSO-SA (20 mg) were dispersed in the mixture solution (water:DMSO = 1:9, v/v), followed by stirring for 3 h. The solution was further dialyzed using a membrane (MWCO 7 kDa, Spectrum Laboratories) against distilled water for 24 h and then filtered through a 0.22 mm pore-sized membrane to remove free PTX and OMP-HAuNS. HP-TCS powder was obtained by lyophilization. The size distribution and zeta potential of HP-TCS micelles were measured using a Zetasizer. The concentration of PTX in the micellar solution was determined by high-performance liquid chromatography (HPLC). For the comparison, OMP-HAuNS and PTX-loaded CSO-SA micelles (HP-CS, non-targeting nanoparticles) were also prepared using a method similar to that described above.

### Cellular competitive uptake

Rhodamine B Isothiocyanate (RITC)-labeled polymer micelles were first prepared by a coupling reaction between the isothiocyanate group of RITC and the amino group of TNYL-CSO-SA micelles in a dark environment at room temperature under magnetic stirring.

EphB4 receptor expression in SKOV3, A549 and CT26 cells was determined by Western blotting. Briefly, cells were treated with cell lysis buffer (Beyotime Institute of Biotechnology, China) containing a protease inhibitor cocktail (Roche Applied Science, Indianapolis, IN) for 30 min at 4 °C. Then, cellular proteins were further extracted by centrifugation under low temperature and added to the loading buffer (Beyotime Institute of Biotechnology, China). The reaction was incubated for 5 min at 95 °C and then cooled. Equal amounts of protein from cells were determined using a BSA Protein Assay Kit (Beyotime Institute of Biotechnology, China) and transferred to a nitrocellulose membrane. EphB4 expression was probed with a mouse anti-EphB4 antibody and an Alexa Fluor 680-conjugated goat anti-mouse IgG (Proteintech, China). Protein bands were visualized with a LI-COR Odyssey system (Lincoln, NE).

Before incubation with polymer micelles, both A549 and NCM460 cells were stained with PKH67 fluorescent cell linker (Sigma-Aldrich; St. Louis, MO), which can incorporate into the cell membrane with no modification of biological activity. Briefly, A549 or NCM460 cells were re-suspended in 500 μL of Diluent C and then 500 μL of PHK67 dye (2 μM) was added. Cells were incubated for 10 min at room temperature. To stop the staining reaction, 1 mL of serum was added and incubated for 2 min followed by centrifugation at 400 × g for 10 min. The cell pellet was washed twice with 6 mL of complete medium to ensure the removal of unbound dye and re-suspended to the desired concentration. PKH67-labeled A549 or NCM460 cells were further co-cultured with SKOV3 or CT26 cells in the same well of a 24-well plate. Then, co-cultured A549/SKOV3 cells or NCM460/CT26 cells were incubated with Rhodamine B isothiocyanate-labeled polymer micelles (RITC: polymer = 2:1, mol/mol) in growth medium for 1 h. In addition, the free TNYL peptide (TNYL:TNYL of HD-TCS = 100:1, mol:mol) was added to the blocking group before incubation with HP-TCS micelles. The final concentration of polymers was 40 μg/mL. After washing the cells with PBS three times, the cellular uptake was observed by confocal laser scanning microscopy (Carl Zeiss LSM 510, Germany). The intensity of cellular fluorescence was further determined using a flow-cytometer (FC500MCL, Beckman Coulter).

### Establishment of a visual orthotopic colon tumor model

All animal studies were performed according to the Institutional Animal Care and Use Committee-approved protocols. Female BABL/C mice (16–18 g; seven weeks of age) were narcotized and fixed on the horizontal flow clean bench with the surgical tape. The abdominal skin of mouse was sterilized using 0.5% (w/v) iodophor and the abdominal cavity was cut longitudinally to reveal normal colorectal tissues. The wound was closed using biodegradable stitches after injecting CT26-Luc cells (∼5 × 10^6^ cells in 20 μL of serum-free DMEM) into the colorectal membrane. Four to eight days later, tumor growth was monitored by observing fluorescent signal using the IVIS Spectrum Imaging System (Caliper,PerkinElmer, USA) after an injection of luciferin (Shanghai Sciencelight Biology Science & Technology Co., Ltd, China). To confirm the orthotopic colon tumors, when the fluorescence reached the acceptable intensity (approximately 1.5 × 10^7^ counts), the mice were killed and the colon tissues bearing tumors were stripped using a medical operation scissor. The tissue was immediately placed into 4% (v/v) formaldehyde solution for hematoxylin-eosin (H&E) and Ki67 staining.

### *In vivo* imaging

Indocyanine green (ICG)-labeled TNYL-CSO-SA was first synthetized by a coupling reaction between the sulfo group of ICG and the amino group of TNYL-CSO-SA micelles in a dark environment at room temperature under the presence of EDC and DMAP, which was used to load HAuNS and PTX for further use.

Female BABL/C mice (16–18 g; seven weeks of age) were inoculated subcutaneously and orthotopicly with CT26 (∼1 × 10^6^ cells in 100 μL of serum-free DMEM) and CT26-Luc cells (∼5 × 10^6^ cells in 20 μL of serum-free DMEM), respectively. When all subcutaneous tumors reached the acceptable sizes or the fluorescence of orthotopic tumors reached the acceptable intensity (approximately 1 × 10^6^ counts), the mice were intravenously injected with ICG-labeled HP-TCS micelles (0.4 mg of polymers in 200 μL of PBS buffer). The mice were observed using the Maestro *In Vivo* Imaging System (CRI Inc., Woburn, MA) or IVIS Spectrum Imaging System at the predetermined time after the injection. At the end of the experiment, the mice were sacrificed. Various tissues, including tumors, were collected, weighted and observed using the *in vivo* imaging system. The fluorescent intensity, which indicates the amount of micelles, was also read using the imaging system. The accumulation of HP-TCS micelles in various tissues was calculated as %ID/g (the percentage of the injected dose per gram of tissue).

As a separate experiment, to investigate the tumor accumulation of HP-TCS mediated by EphB4 receptors, paired tumor models were generated by subcutaneous injection of SKOV3 and A549 cells (5 × 10^6^ cells) into the left and right sides of female nude mice (18–22 g, 6–8 weeks of age), respectively. The mice were intravenously injected with ICG-labeled HP-TCS micelles (0.4 mg polymers). The biodistribution of the micelles was investigated by measuring the fluorescence at the predetermined time using an *in vivo* imaging system.

### *In vivo* immunohistochemistry analysis

The expression of EphB4 receptor in SKOV3, A549 and CT26 tumors was determined by immunohistochemistry staining and the intratumoral distribution of HP-TCS micelles was also investigated. Briefly, mice bearing SKOV3, A549 or CT26 tumors were injected intravenously with ICG-labeled HP-TCS (ICG-HP-TCS). After 24 h, the mice were sacrificed and the tumor tissues were collected. Then, the tumors were immediately embedded in tissue-tek OCT and cut into 5 µm slices for staining of the endothelial marker CD31 (PECAM-1) and EphB4 antibody (Anti-EphB4). The EphB4 receptor expression and the intratumoral distribution of HP-TCS in three tumors were detected by confocal laser scanning microscopy (IX81-FV1000, Olympus Corporation, Japan).

### *In vivo* anti-tumor activity

The *in vivo* anti-tumor activity of HP-TCS was investigated in subcutaneous SKOV3 or CT26 tumor models and the orthotopic CT26-Luc tumor models. When all subcutaneous tumors reached the acceptable sizes (about 10 days, approximately 100–150 mm^3^) or the fluorescence of orthotopic tumors reached the acceptable intensity (about a week, approximately 1 × 10^6^ counts), the mice were intravenously injected with agents. For the subcutaneous SKOV3 tumors, female nude mice bearing the tumors were randomly allocated into six groups. The mice in groups 1 to 6 were injected intravenously each time with saline (*n* = 6), free PTX (*n* = 6, 5 mg PTX/kg body weight), P-TCS (PTX-loaded TCS, *n* = 6, 5 mg PTX/kg body weight), H-TCS (HAuNS-loaded TCS, 3 × 10^14^ particles/kg body weight), HP-CS (non-targeting HAuNS and PTX-loaded CS, *n* = 6, 5 mg PTX/kg and 3 × 10^14^ particles/kg body weight) and HP-TCS (targeting HAuNS and PTX-loaded TCS, *n* = 6, 5 mg PTX/kg and 3 × 10^14^ particles/kg body weight), respectively. For the treatment of subcutaneous CT26 tumors, female BABL/C mice bearing the tumors were randomly allocated into four groups. The mice in groups 1 to 4 were injected intravenously each time with saline (*n* = 8), free PTX (*n* = 8, 5 mg PTX/kg body weight), HP-CS (*n* = 8, 5 mg PTX/kg and 3 × 10^14^ particles/kg body weight) and HP-TCS (*n* = 8, 5 mg PTX/kg and 3 × 10^14^ particles/kg body weight), respectively. All mice were injected for a total three times on days 1, 3 and 5 (one injection for 1 day) and all tumors in mice were irradiated with an NIR laser (1.0 W/cm^2^ for 2 min) for a total of three times on days 2, 4 and 6 (one irradiation for one day). The body weight of each mouse was monitored every two days. Tumor growth was determined by measuring three orthogonal tumor diameters. Tumor volume was calculated using the formula π × abc/3, where a, b and c are the length, width and height diameters of a tumor, respectively. The experiment was terminated on day 21 after initial treatment or when tumors in the control group reached >1000 mm^3^ or >3000 mm^3^ (CT26 tumors). At the end of the experiment, the mice were sacrificed and the tissues, including tumor, heart, liver, spleen, lung and kidney, were collected. To study the toxic effects of HP-TCS micelles on organs, the tissue sections were embedded in paraffin and further identified by Ki-67 immunostaining and H&E staining. The samples were then observed using a fluorescence microscope.

For the orthotopic CT26-Luc tumors, female BABL/C mice bearing tumors were randomly allocated into three groups. The mice in groups 1 to 3 were intravenously injected each time with saline (*n* = 3), free PTX (*n* = 3, total 20 mg PTX/kg body weight), HP-CS and HP-TCS (*n* = 3, total 20 mg PTX/kg and 12 × 10^14^ particles/Kg body weight), respectively. The mice were injected for a total of four times on days 1, 3, 5 and 7 (one injection for 1 day). A BioTex LCM-001 optical fiber was used to deliver the laser to the target. All tumors in mice were precisely irradiated with an NIR laser for a total of four times on days 2, 4, 6 and 8 (one irradiation for one day) (1.0 W/cm^2^ for 1 min) under high-resolution B-mode ultrasound guidance (Philips IU 22, NL). The body weight of each mouse was monitored every two days. Tumor growth was monitored each week by measuring the tumor fluorescence intensity using the IVIS Spectrum Imaging System. The experiment was terminated on day 21 after initial treatment.

At the end of experiment, all mice were sacrificed, and the tissues, including tumor, heart, liver, spleen, lung and kidney, were collected for Ki67 and H&E staining.

### Data collection and analysis

Statistically significant differences between pairs of mean values were determined with ANOVA followed by Tukey- Kramer tests. Differences between the groups were analyzed with Student’s *t*-test, and *p* values of less than .05 were deemed statistically significant. All the statistical analyses were conducted using Microsoft Excel software.

## Results

### Preparation and characteristics of HP-TCS nanoparticles

The synthesis of TNYL-CSO-SA micelles was confirmed using the ^1^H NMR spectrum (Supplementary Figure S1). The encapsulated efficiency of OMP-HAuNS and PTX in TCS was greater than 80% according to our previously reported method (You et al., 2013) (Supplementary Table S1). The final HP-TCS nanoparticles still exhibited a typical surface plasmon resonance peak in the NIR region (∼800 nm) (Supplementary Figure S2). HP-TCS presented a specific Core/Shell structure, where the OMP-modified HAuNS served as a core and the TNYL-conjugated CSO-SA polymer served as the shell (Supplementary Figure S3), which was confirmed by TEM imaging (Supplementary Figure S4). Due to the encapsulation of OMP-HAuNS, the mean diameter of HP-TCS increased significantly from 58.2 ± 3.0 (TCS, blank micelles) to 68.6 ± 5.4 nm, which was determined using the dynamic light scattering method (Supplementary Figure S5). The surface potential of HP-TCS was 32.1 ± 1.3 mV, with no significant difference compared with TCS.

### *In vitro* cellular competitive uptake studies

**Figure 1.**
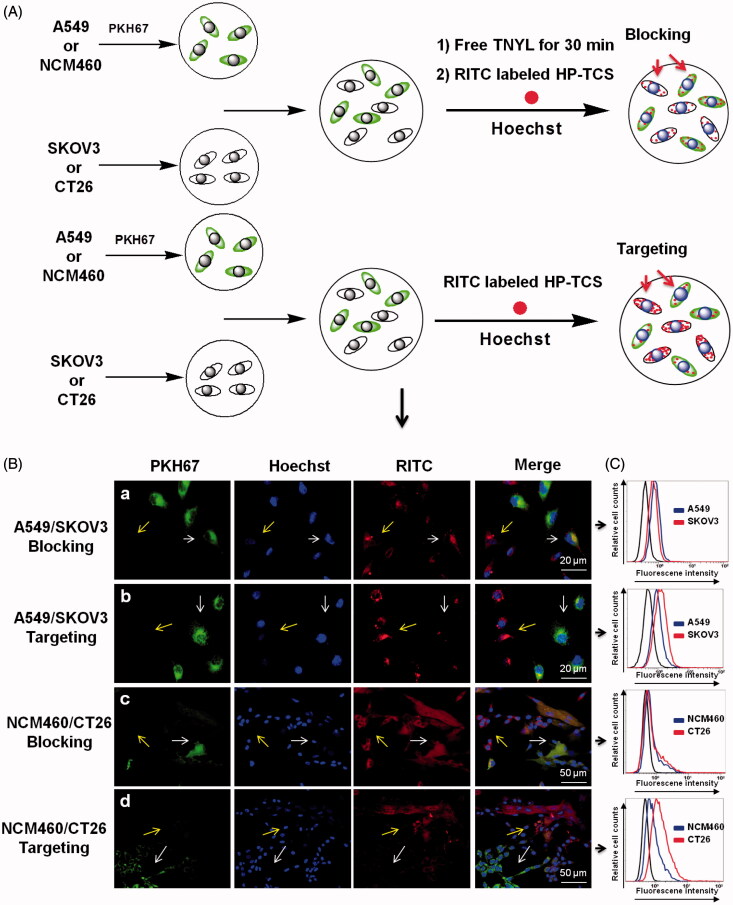
Cellular competitive uptake studies. Confocal microscopy images of the cellular competitive uptake of RITC-labeled nanoparticles for 1 h (the cells were all stained with Hoechst 33342). (A) Schematic illustration of cellular competitive uptake. (B) Confocal microscopy images of the cellular competitive uptake of RITC-labeled HP-TCS nanoparticles after 1 h of incubation. All cells were stained with Hoechst 33342. A549 and NCM460 cells were labeled with PKH67 fluorescent linker and co-cultured with SKOV3 and CT26 cells, respectively. Then, the cells were incubated with HP-TCS and HP-TCS plus free TNYL. Scale bar: 50 µm. Quantitative uptake in A549 cells and SKOV3 or NCM460 cells and CT26 for this co-cultured system was measured using a flow-cytometer.

The expression of EphB4 receptor in NCM460, CT26, SKOV3 and A549 cells was investigated by Western blotting. CT26 and SKOV3 exhibited high EphB4 expression, whereas NCM460 and A549 exhibited low expression (Supplementary Figure S6). To evaluate the specific cellular uptake of HP-TCS, the co-cultured systems containing EphB4-positive and EphB4-negative cells were generated by incubating SKOV3 and A549 cells or CT26 and NCM460 cells in the same wells followed by further incubation with our micelles (Figure 1(A)). As shown in Figure 1(B), a significant difference in the cellular internalization of HP-TCS was noted in the co-cultured system containing SKOV3/A549 or NCM460/CT26 cells. The increased cellular uptake of HP-TCS to SKOV3 cells (high EphB4 receptor expression, yellow arrows) was noted compared with A549 cells (low EphB4 receptor expression) during a short incubation time (i.e. 1 h). Similar results were also demonstrated when HP-TCS was incubated with the co-culture system of NCM460 and CT26 cells. EphB4-positive CT26 cells exhibited increased HP-TCS internalization compared with EphB4-negative NCM460 cells. The uptake of HP-TCS exhibited no significant difference between EphB4-positive and EphB4-negative cells after free TNYL peptide was added to the co-cultured system of SKOV3/A549 cells or NCM460/CT26 cells (Figure 1(B)).

Under the same conditions, further quantitative analysis of the cellular competitive uptake of the micelles in the SKOV3/A549 and NCM460/CT26 cell co-cultured system was performed using flow cytometry (Figure 1(C)). The average fluorescent intensity, which indicates the amount of HP-TCS cellular internalization in SKOV3, A549, CT26 and NCM460 cells was 7.86, 1.44, 4.06 and 0.918, respectively, indicating the increased cellular internalization of the micelles into EphB4-positive cells compared with EphB4-negative cells.

After free TNYL peptide was added to the co-culture system, the fluorescence values in SKOV3, A549, CT26 and NCM460 cells were 1.35, 1.26, 0.955 and 0.824, respectively. These results were consistent with those observed by confocal laser scanning microscopy (Figure 1(B)). Our data demonstrated that HP-TCS could be specifically internalized by EphB4-positive cells via receptor-mediated endocytosis.

### *In vitro* selective cell killing ability

An EphB4-mediated cancer cell killing effect was investigated by incubating the co-culture system of SKOV3/A549 cells with HP-TCS followed by the measurement of cell survival. The selective cell killing ability of HP-TCS was evaluated by the analysis of the relative SKOV3:A549 ratio.

As shown in Supplementary Figure S7, after co-cultured cells were incubated with free PTX for 48 h (as the positive control group), the relative cell ratio of A549:SKOV3 was 52.6:47.4, which was not significantly different when compared with (55.5:44.5) the negative control group (no treatment). However, the A549:SKOV3 ratio was 68.2:31.8 when the co-cultured cells were incubated with HP-TCS for 48 h, indicating increased SKOV3 cell death in the co-culture system.

The results suggested a very high correlation between cell death and the binding of TNYL peptide with EphB4 receptors. With the modification of the TNYL peptide, more HP-TCS were selectively internalized into EphB4-positive SKOV3 cells, thus resulting in increased cell death in specific cell types. In contrast, free PTX exhibited no selectivity for the EphB4 receptor, causing similar cell death between SKOV3 and A549 cells.

### Targeting accumulation into orthotopic tumors

To investigate the efficiency of the accumulation of HP-TCS in orthotopic tumors, a visual orthotopic colon tumor model was established by injecting CT26-Luc cells with the expression of insect luciferase into the colorectal membrane after abdominal surgery. Strong bioluminescence was observed on the abdomen under the IVIS Spectrum Imaging System at 4–8 days after the surgery (Supplementary Figure S8(A)). To further confirm the orthotopic tumor model, representative mice were dissected and the colon tissue was collected. Tumor cells connective to normal colon were confirmed by H&E and Ki67 staining (Supplementary Figure S8(B)), exhibiting large nuclei and increased proliferation.

**Figure 2.**
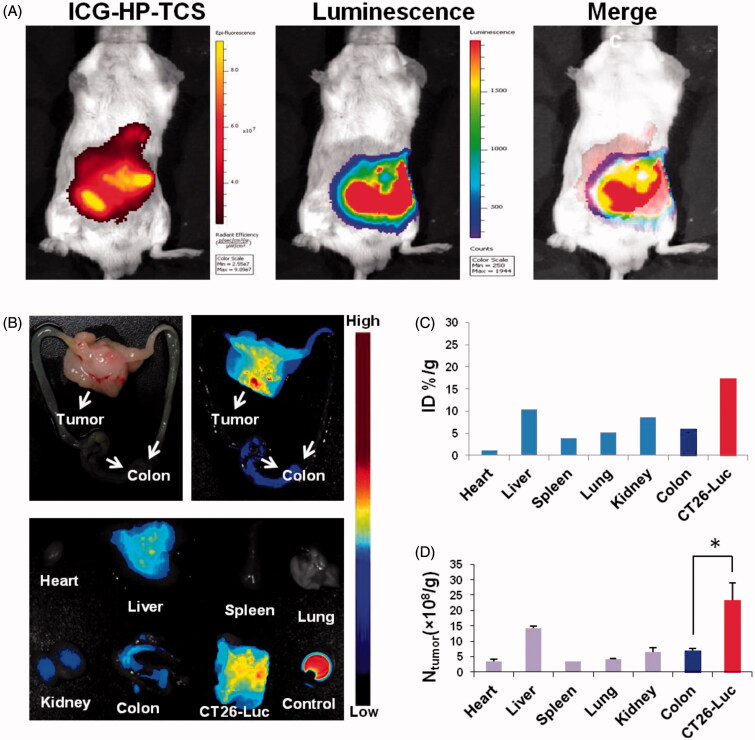
Bioimaging studies in orthotopic models. (A) The *in vivo* imaging of ICG-HP-TCS in orthotopic CT26-Luc tumor-bearing BABL/C mice model at 5 min after IP injection of D-luciferin. (B) The photograph and *in vivo* imaging of CT26-Luc tumor, normal colon and various tissues at 24 h after the intravenous injection of ICG-HP-TCS nanoparticles. (C) The accumulation of ICG-HP-TCS nanoparticles in various tissues was calculated as %ID/g (the percentage of the injected dose per gram of tissue). The fluorescent intensity, which indicates the amount of micelles, was read by the imaging system. (D) The accumulation of ICG-HP-TCS nanoparticles in various tissues by measuring Au element levels in tumors using ICP-MS (*n* = 3). *p < .05.

After the injection of ICG-labeled HP-TCS for 24 h, strong ICG fluorescence was colocalized with bioluminescence, suggesting the high accumulation of our micelles in the orthotopic tumors (Figure 2(A)). Then, the colons that harbored tumors were further collected. Strong ICG fluorescence was noted in the tumors, whereas weak fluorescence was noted in normal colon tissue (Figure 2(B)). Approximately 3-fold increased accumulation of the micelles was observed in tumors (%ID/g = 17.33) compared with the surrounding normal colon (%ID/g = 6.75) as assessed by fluorescence intensity (Figure 2(C)). More persuasive data were obtained by measuring the amount of Au in various tissues using ICP-MS. Significantly increased Au distribution was noted in tumors compared with normal colon (greater than 4-fold increased) and other organs (including heart, liver, spleen, lung and kidney) (Figure 2(D)). The data demonstrated that HP-TCS exhibits remarkable targeting delivery into orthotopic colon tumor and this effect could be attributed to the increased expression of EphB4 receptors in the tumors compared with normal colon tissue. As a comparison, an ectopic colon tumor model was also established by subcutaneously injecting CT26-Luc cells and a similar result was obtained after injecting ICG-labeled HP-TCS, indicating the co-localization of ICG fluorescence from the micelles with bioluminescence from the CT26 tumors (Supplementary Figure S9). A gradual increase in the accumulation of the micelles in the subcutaneous tumors was demonstrated from 1 to 48 h after injection (Supplementary Figure S9(B)).

Interestingly, the increased accumulation of HP-TCS was observed in orthotopic tumors compared with ectopic tumors at 48 h after injection (Figure 2(B–D)) possibly because orthotopic colon tumors develop a more advanced microvasculature than subcutaneous tumors, inducing the more efficient accumulation of our micelles in the orthotopic tumors. The highly selective delivery of HP-TCS into orthotopic or atopic colon tumors offers safer and more effective tumor treatment. A paired tumor model containing EphB4-positive (SKOV3) and EphB4-negative (A549) tumors were further employed to investigate the EphB4-mediated tumor delivery of HP-TCS. Increased fluorescence was observed in SKOV3 tumors when compared with A549 tumors throughout the entire experimental process (Supplementary Figure S10), which is attributed to increased EphB4 expression in SKOV3 cells when compared with A549 cells.

**Figure 3.**
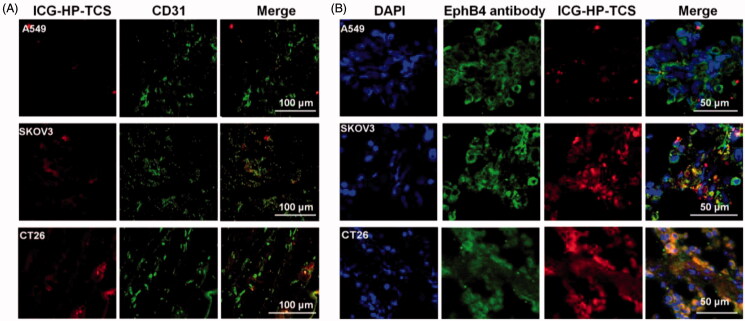
Immunohistochemical staining studies. (A) Immunohistochemical staining of ICG-HP-TCS nanoparticles using CD31 antibody in SKOV3, A549 and CT26 tumor vessels. (B) Immunohistochemical staining of ICG-HP-TCS micelles using EphB4 antibody in SKOV3, A549 and CT26 tumors. Nuclei were counterstained with 4,6-diamidino-2-phenylindole.

The results were consistent with those in our previous reports (Wang et al., 2015), where the accumulation of the micelles in SKOV3 tumors was obviously inhibited after pre-injection of free TNYL peptide to block EphB4 receptors in the tumor cells, suggesting specific tumor delivery of the micelles mediated by EphB4 receptors. Immunohistochemical staining results from SKOV3, A549 and CT26 tumors using EphB4 and a CD31 antibody revealed that significantly increased HP-TCS accumulated in SKOV3 and CT26 tumors that overexpress the EphB4 receptor when compared with A549 tumors with low EphB4 expression (Figure 3). HP-TCS could further extravasate microvessels into deeper tumor tissue (Figure 3(A)) and exhibit co-localization with the EphB4 receptor in the tumor cells, indicating specific binding of micelles with the receptor (Figure 3(B)).

The results demonstrate the feasibility of increasing the accumulation of our micelles in EphB4-positive tumors via the interaction between the TNYL-peptide on the micelles and EpHB4 on tumor cells and indicate that HP-TCS has specific targeting ability for orthotopic or atopic tumors with high EphB4 expression.

### *In vivo* anti-tumor activity studies

We verified the NIR laser-thermal conversion efficiency of HP-TCS *in vitro* and *in vivo* (Supplementary Figure S11(A,B)).

A greater than 30 °C increase in the aqueous solution containing HP-TCS (0.5 mg/mL) was noted under the continuous exposure of a NIR laser (output power of 1.5 W) for 10 min *in vitro*. In addition, a greater than 20 °C temperature change was noted in CT26 tumor tissues after an intravenous injection of HP-TCS (15 mg/kg PTX and 9 × 10^14^ particles/Kg HAuNS) under continuous exposure to the NIR laser (output power of 1 W) for 5 min. The *in vivo* bio-safety of NIR laser irradiation was also investigated by irradiating externally and subcutaneously on the healthy skin of mice after the intravenous injection of HP-TCS (15 mg/kg PTX and 9 × 10^14^ particles/Kg HAuNS), followed by a histology examination of the irradiated area.

**Figure 4.**
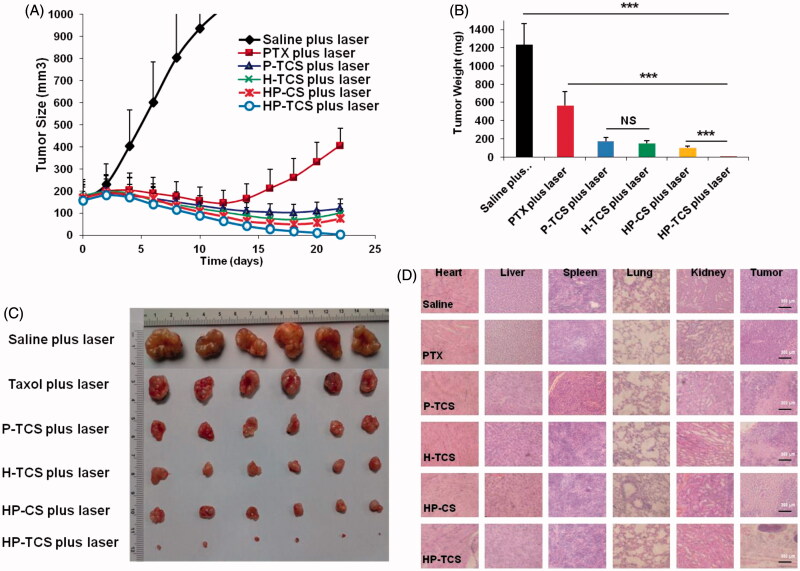
*In vivo* anti-tumor activity studies in subcutaneous SKOV3 tumor models. (A) Tumors growth curves for the mice bearing SKOV3 tumors after the intravenous injection of saline, PTX, H-TCS, P-TCS, HP-CS and HP-TCS nanoparticles. (B) The average tumor weights in different treatment groups. (C) Representative photographs of tumors in saline plus laser, PTX plus laser, P-TCS plus laser, H-TCS plus laser, HP-CS plus laser and HP-TCS plus laser groups after irradiation treatment. (D) Histologic evaluation of tumor tissues and various organs in mice treated with each treatment group after NIR laser treatment and stained with H&E. Data represent the mean ± standard deviation (*n* = 6). *p < .05, **p < .01 and ***p < .005.

The results indicated that irradiation with <2 W/cm^2^ output power and a < 4 min duration was feasible and safe (Supplementary Figure S12). Then, the *in vivo* anti-tumor activity of HP-TCS was first evaluated in the implanted subcutaneous SKOV3 and CT26 tumor-bearing mouse models. The curves of SKOV3 tumor growth were observed for all the tested groups (Figure 4(A)). When compared with the control group (the average initial size is 159.03 mm^3^) after treatment, the tumor growth was significantly inhibited after intravenous injections of free PTX (15 mg/kg PTX in total), P-TCS (15 mg/kg PTX in total), H-TCS (9 × 10^14^ particles/Kg HAuNS in total), HP-CS (15 mg/kg PTX and 9 × 10^14^ particles/Kg HAuNS in total as non-targeting micelles) and HP-TCS (15 mg/kg PTX and 9 × 10^14^ particles/Kg HAuNS in total as targeting micelles) followed by NIR laser irradiation (1 W/cm^2^ output power for 2 min for each time for a total of three times). The HP-TCS-plus-laser presented the strongest antitumor activity, inducing significantly smaller mean tumor weight (5.0 ± 3.9 mg (*n* = 6) after three-week treatment when compared with saline-plus-laser (1235.2 ± 232.9 mg on day 12, *n* = 6; *p* < .0001), free PTX-plus-laser (566.7 ± 148.6 mg, *n* = 6; *p* < .0001), P-TCS-plus-laser (173.9 ± 39.9 mg, *n* = 6; *p* < .0001), H-TCS-plus-laser (148.5 ± 27.3 mg, *n* = 6; *p* < .0001) and HP-CS-plus-laser (100.7 ± 17.2 mg, *n* = 6; *p* < .001) (Figure 4(B,C)) treatments. The tumors of the six mice in the HP-TCS-plus-laser treatment group almost disappeared or became small scar tissues at the end of experiment based on external observation.

Histological analysis indicated serious damage to remaining tumor tissues after HP-TCS-plus-laser treatment, whereas the other organs appeared normal similar to those in the saline group (Figure 4(D)).

**Figure 5.**
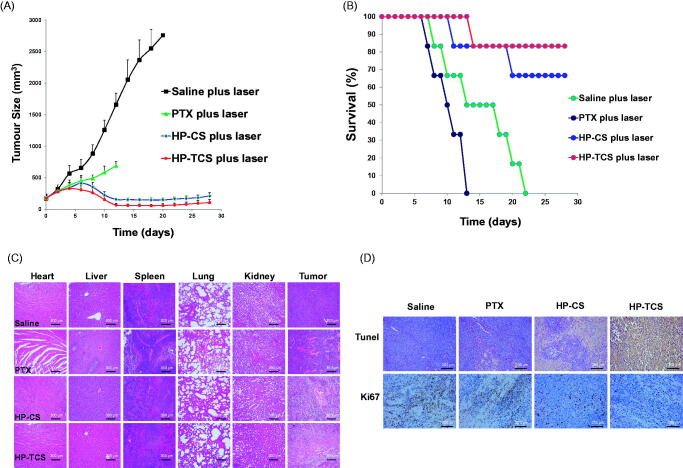
*In vivo* anti-tumor activity studies in subcutaneous CT26 tumor models. (A) Tumor growth curves for BABL/C mice bearing CT26 tumors after the intravenous injection of saline, PTX, HP-CS and HP-TCS nanoparticles, irradiated by NIR laser (1.0 W for 3 min) at 24 h. (B) The survival rate curves of BABL/C mice in saline plus laser, PTX plus laser, HP-CS plus laser and HP-TCS plus laser groups after irradiation treatment. (C) Histologic evaluation of tumor tissues and various organs in BABL/C mice treated with each treatment group at 12 days after NIR laser treatment and stained with H&E. (D) Immunological staining evaluation of tumor tissues in mice treated with each treatment group at 12 days after NIR laser treatment and stained with TUNEL and Ki67. Data represent the mean ± standard deviation (*n* = 6).

The remarkable antitumor effect of HP-TCS under the irradiation of the NIR laser is attributed to the strong photothermal-chemotherapy combination. The mean body weight of mice in the free PTX-plus-laser, HP-CS-plus laser and HP-TCS-plus laser groups significantly decreased from 0 to 8 days after the first injection (Supplementary Figure S13) but gradually recovered in the subsequent stage, inducing an increased weight at the end of experiment compared with that on day 0. This finding suggested that the systemic toxicity of the treatments could be low or eliminated by extending the time. Figure 5(A) presents subcutaneous CT26 tumor growth profiles after the injections of saline, free PTX (15 mg/kg PTX, in total), HP-CS (15 mg/kg PTX and 9 × 10^14^ particles/Kg HAuNS, in total) and HP-TCS (15 mg/kg PTX and 9 × 10^14^ particles/Kg HAuNS, in total). The results revealed that HP-TCS-plus-laser exhibited the strongest antitumor activity when compared with other treatments, inducing the longest survival for the mice in the group (Figure 5(B)). The mean tumor volume in the HP-TCS-plus-laser group on day 12 was 67.45 ± 21.02 mm^3^ (*n* = 8), which was significantly reduced when compared with the saline-plus-laser group (1658.81 ± 309.56 mm^3^, *n* = 8; *p* < .0001) and the HP-CS-plus-laser group (156.73 ± 25.42 mm^3^, *n* = 8; *p* < .001). Although the free PTX-plus-laser group also exhibited some tumor inhibitory effect (586.12 ± 70.25 mm^3^, *n* = 8, *p* < .01) when compared with the saline-plus-laser group, almost all of the mice were dead after 12 days of treatment and suffered severe weight loss (Supplementary Figure S13(B)), indicating the significant potential systemic toxicity of free PTX. Spleen and livers in the free PTX-plus-laser group exhibited obvious damage, whereas the damage was not significant for these organs in other groups (Figure 5(C)). In addition, TUNEL and Ki67 staining of tumors confirmed that the HP-TCS-plus-laser could induce increased apoptosis in CT26 tumor cells (Figure 5(D)).

**Figure 6.**
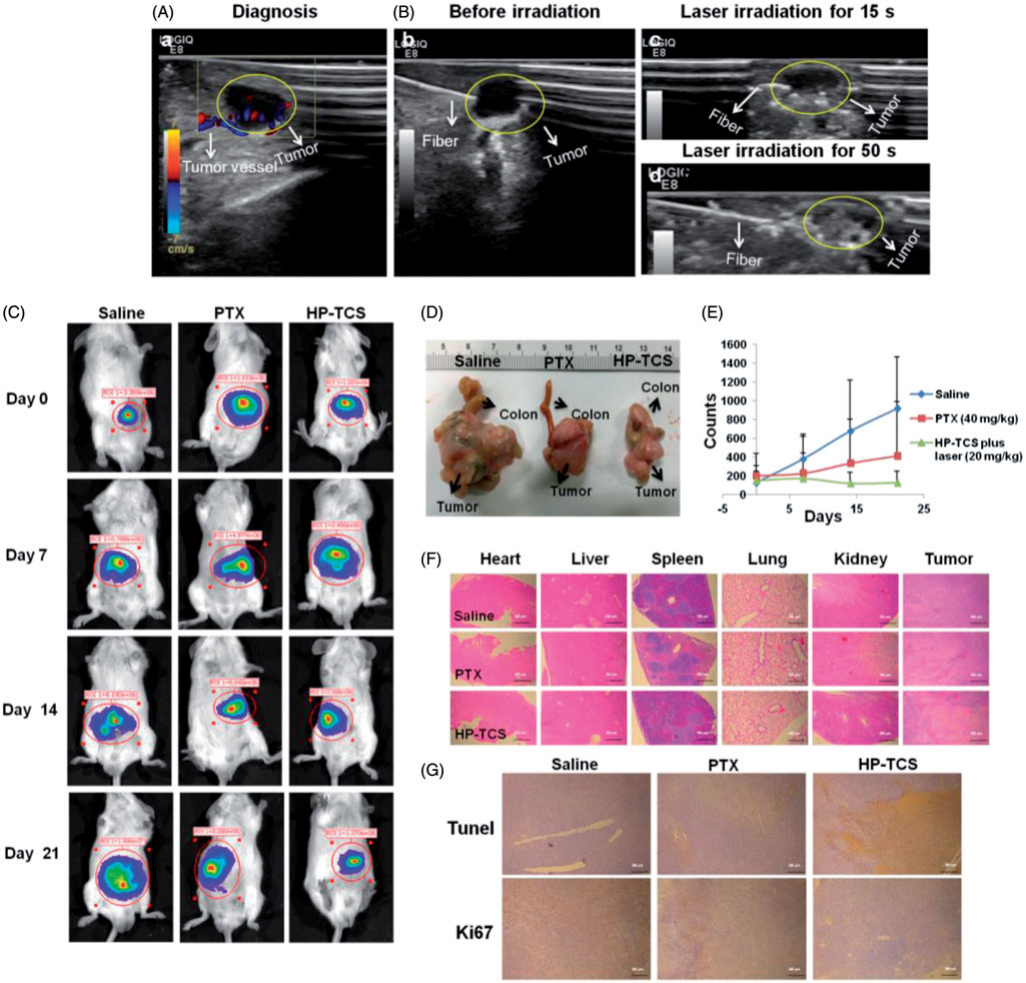
The *in vivo* anti-tumor activity studies in orthotopic CT26-Luc tumor models. (A) B mode ultrasound images of the orthotopic CT26-Luc tumor model, (a) diagnosis, (B) before irradiation (b), laser irradiation for 15 s (c) and laser irradiation for 50 s (d). (C) The *in vivo* imaging of BABL/C mice bearing orthotopic CT26-Luc tumors at 5 min after IP injection of D-luciferin treated with saline, PTX and HP-TCS-plus-laser under the guidance of B mode ultrasound after 0, 7, 14 and 21 days. (D) Post-autopsy representative image of CT26-Luc tumor and colon, treated with saline, PTX and HP-TCS-plus-laser at 21 days. (E) Tumor growth curves for bearing CT26-Luc tumors BALB/C mice of ‘B’; the luminescence counts were read by the *in-vivo* imaging systems. (F) H&E staining of CT26-Luc tumor tissues and various organs in BABL/C mice, treated with each treatment group at 12 days after NIR laser treatment. (G) TUNEL and Ki67 staining of CT26-Luc tumor tissues in mice, treated with each treatment group at 12 days after NIR laser irradiation. Data represent the mean ± standard deviation (*n* = 3).

Next, an orthotopic CT26 tumor model with luciferase expression was employed to test the photothermal-chemotherapy effect of HP-TCS. The *in vivo* imaging of implanted CT26-Luc tumors by injecting different cell amounts showed that the fluorescence intensity of the tumors was consistent with the number of cells (Supplementary Figure S14). The injection dose of HP-TCS was increased to 20 mg/kg which was equivalent to PTX and 1.2 × 10^15^ particles/Kg HAuNS in total, but the irradiation duration was reduced to 1 min under 1 W/cm^2^ output power to guarantee that the irradiation was safe for normal tissues close to the orthotopic tumors. Precise irradiation was obtained by transferring the NIR laser to the center of the tumor using an optical fiber under the guidance of high-resolution B-mode ultrasound imaging. The outline of the CT26-Luc tumor (the shaded region) and inserted fiber was clearly visible, and the fast-flowing blood signal in the tumor was also observed under ultrasound imaging (Figure 6(A)).

Based on the ultrasound imaging, the irradiation of NIR laser caused obvious photothermal effect in tumor region after the injection of HP-TCS, inducing the rapid ablation of the tumors (Figure 6(B)). As shown in Figure 6(E), tumor growth in the saline-plus-laser group was almost out of control, all of these tumors grew rapidly. In contrast, although tumor growth in the PTX-plus-laser group was inhibited, these tumors still were slowly growing. However, tumor growth in the HP-TCS-plus-laser group was significantly inhibited as assessed by the analysis of mean bioluminescence counts from 149 on day 0 to 124 on day 21, whereas the mean bioluminescence counts of the tumors in the saline-plus-laser group were 916 on day 21 with a greater than 6-fold increase when compared with day 0 (Figure 6(C–E)). The stronger efficacy of the HP-TCS-plus-laser was also confirmed by histological analysis (Figure 6(F,G)). The mean body weight of mice in the HP-TCS-plus-laser group increased during the entire experiment (Supplementary Figure S15), suggesting low systemic toxicity with an increased dose.

## Discussion

Polymeric micelles are promising nanocarrier systems for antitumor drug delivery, which might be one effective method to improve antitumor efficiency and reduce systemic toxicity caused by chemotherapy. Recently, combined cancer photothermal-chemotherapy based on gold nanoparticles has received increasing attention (Banu et al., 2015; Zhang et al., 2013). However, the potential toxicity and instability of gold nanoparticles-bearing chemotherapeutic agents cannot be belittled (Siddiqi et al., 2012; Caballero-Díaz E & Valcárcel M, 2014). To solve the problem, we used an amphiphilic glycolipid-like chitosan-stearic acid copolymer (CSO-SA), which was synthesized by the conjugation between chitosan oligosaccharide and stearic acid that displayed a so-called special spatial structure of multiple ‘minor-cores’ in the aqueous medium with excellent internalization into cells and increased cellular uptake of the payload, to encapsulate HAuNS and PTX (Supplementary Figure S3). To improve the tumor-specific delivery of the micelles, a targeted TNYL peptide with specific binding to EphB4 receptors was conjugated onto the surface of the micelles to increase tumor-selective accumulation. To obtain more effective treatment outcomes in cancer, it is crucial that drugs can be efficiently delivered to the targets and subsequently exhibit rapid release in the region. Our drug delivery system exhibited time- and space-controlled drug release from polymeric micelles based on a photothermal effect triggered by NIR light. The triggered-release of PTX from the established system responding to the turn-on of a NIR laser was feasible in our recent study (You et al., 2013), indicating that it was possible for the entrapped drug to present a temporal and spatial control release by a local NIR laser irradiation. Then, chemotherapy combined with photothermal ablation treatment for cancer is provided. Therefore, it is necessary to further evaluate the *in vivo* anti-tumor activity and the tumor targeting efficiency of our system.

Given that almost all the reported strategies based on photothermal-chemotherapy have exclusively focused on the treatment for superficial or subcutaneous cancer (Zhang et al., 2016), our system was mainly used to treat deep orthotopic tumors using the NIR laser. In this study, we sought to assess the feasibility of photothermal combined treatment for deep orthotopic tumors based on our system. Regarding the safety and high efficiency of the treatment, it is important that our micelles exhibit highly specific accumulation in the orthotopic tumors but not in normal tissues surrounding the tumors, inducing a large concentration gradient of the micelles between the tumor and the normal tissues. Thus, the irradiation will cause photothermal ablation only against the tumors and will not harm normal tissues. Then, the specific delivery of the micelles into the tumors mediated by the EphB4 receptors was investigated *in vitro* and *in vivo*. First, two co-cultured systems (SKOV3/A549 and CT26/NCM460 cells) containing EphB4-positive and EphB4-negative cells were established and incubated with our micelles, revealing the specific binding and the selective killing of the micelles to EphB4-positive cells (Figure 1 and Supplementary Figure S7). Next, the targeting delivery of the micelles into orthotopic and subcutaneous colon tumors were verified after intravenous injection. Interestingly, our micelles exhibited more specific accumulation in orthotopic tumors compared with subcutaneous tumors (Figure 3 and Supplementary Figure S9), which was possibly attributed to the more advanced microvasculature in orthotopic colon tumors compared with the subcutaneous tumors. The biodistribution study in mice bearing an EphB4-positive tumor on one side and an EphB4-negative tumor the other side also indicated significantly more retention of the micelles in the tumors with high EphB4 expression (Supplementary Figure S10). These data demonstrated that HP-TCS are selectively distributed into EphB4-positive orthotopic or ectopic tumors via the specific binding of the TNYL peptide with EphB4 receptors.

The orthotopic colon tumors could be irradiated precisely with an NIR laser using an optical fiber under the guidance of ultrasound imaging. After analyzing the damage of healthy mouse skin under various NIR laser irradiation conditions, irradiation with 1 W/cm^2^ for 1 min duration does not harm normal tissues (Supplementary Figure S12). However, an obvious photothermal ablation effect could be rapidly caused within the tumors under the conditions, which is attributed to the highly specific accumulation of our micelles in the tumors. The remaining tumor cells are exposed to chemotherapy due to a triggered release of PTX from the micelles via a photothermal effect. As a result, HP-TCS combined with NIR laser irradiation exhibited increased antitumor efficacy with low systemic toxicity (Figure 6). Similar results were obtained when the subcutaneous SKOV3 or CT26 tumors were treated with the micelles and laser (Figures 4 and 5).

## Conclusions

In this study, we prepared an EphB4 receptor-targeting polymeric nanoplatform containing HAuNS and PTX for combined cancer photothermal-chemotherapy. This work demonstrated the feasibility using a targeted peptide to increase tumor targeting. With the modification of the TNYL peptide, HP-TCS could specifically internalize into EphB4-positive SKOV3 and CT26 cells, further inducing the selective killing of the cells in a co-culture system simultaneously containing EphB4-positive and EphB4-negative cells. Targeting the micelles into implanted orthotopic or subcutaneous tumors with high EphB4 expression was observed using *in vivo* fluorescence and bioluminescence imaging systems. Interestingly, increased HP-TCS accumulation was observed in orthotopic colon tumors compared with ectopic tumors. Highly specific accumulation of HP-TCS in EphB4-positive tumors significantly increased the feasibility of photothermal-chemotherapy mediated by NIR laser. Then, a systemic study of antitumor activity was performed in various tumor models, including implanted subcutaneous and visual orthotopic tumors. Precise NIR laser irradiation to the tumors was obtained under the guidance of high-resolution B-mode ultrasound imaging, causing a rapid photothermal ablation effect limited to the tumor region. Tumor growth was significantly inhibited by the combination of photothermal therapy and chemotherapy due to the triggered release of PTX. Our study provided a promising strategy of NIR laser-mediated photothermal-chemotherapy based on HP-TCS against tumors (especially, deep orthotopic tumors) with high EphB4 expression.

## Supplementary Material

IDRD_You_et_al_Supplemental_Content.doc
