# Supplementary material for: Near infrared light mediated photochemotherapy for efficiently treating deep orthotopic tumors guided by ultrasound imaging
Source: Drug Deliv. 2017 Sep 25;24(1):1441–52. doi: 10.1080/10717544.2017.1375574 (PMC8241057; doi:10.1080/10717544.2017.1375574)
Supplement: IDRD_You_et_al_Supplemental_Content.doc [file IDRD_A_1375574_SM4618.doc]

**Supporting Information**

Supplementary Methods, Supplementary Figures and legends

This file contains Supplementary Figures S1-S15 and Supplementary Table 1 and Supplementary Methods 1-4

Supplementary Figure S1

**
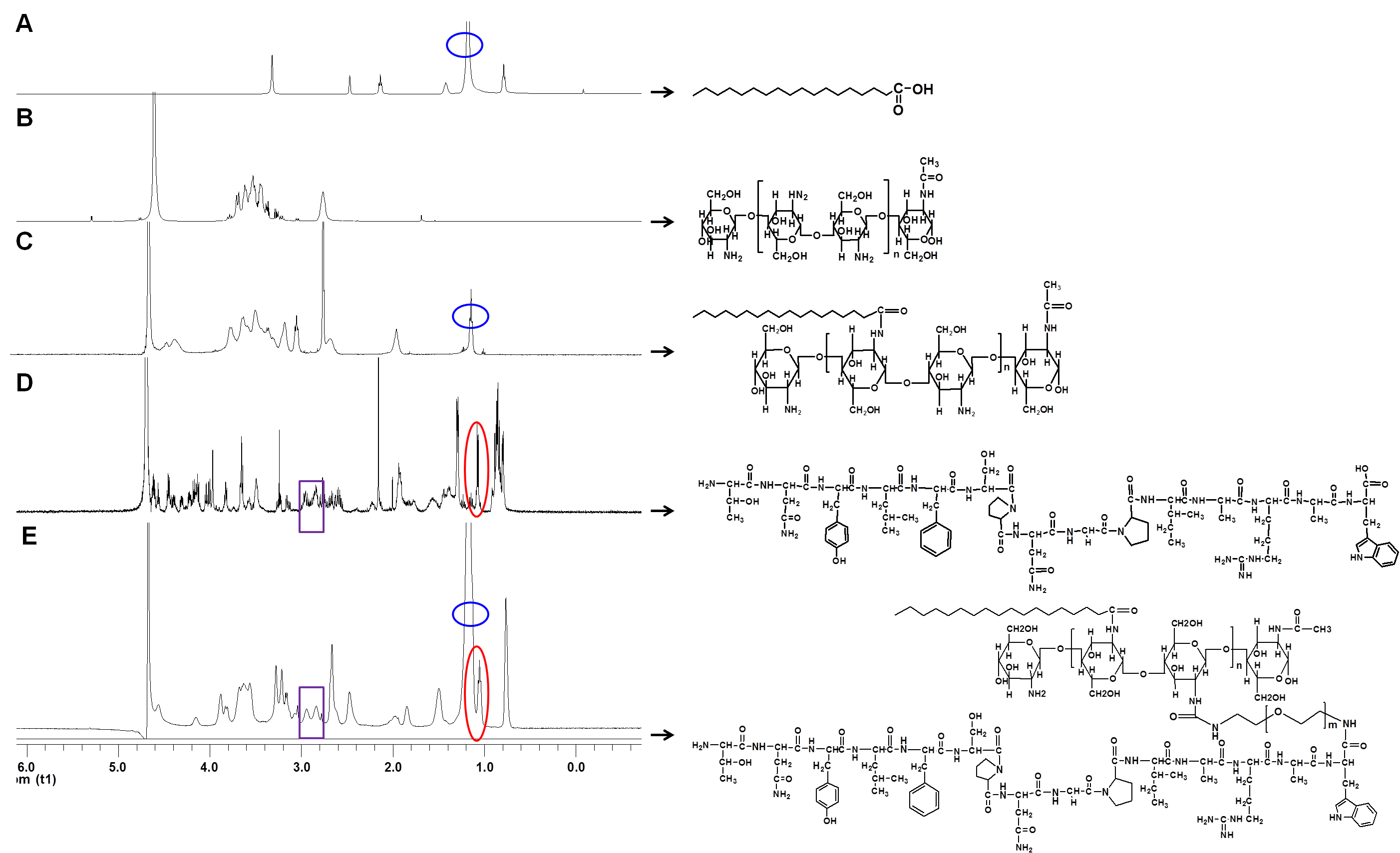
**

**Supplementary Figure S1.** ^1^H NMR spectra of stearic acid (SA, **A**), chitosan **(**CSO, **B**), chitosan-stearic (CSO-SA**, C**), TNYL peptide (**D**) and TNYL polypeptide conjugated CSO-SA micelles (TNYL-CSO-SA, **E**). The final TNYL-CSO-SA has appeared the characteristic peak signal, that's completely consistent with other monomers. ^1^H NMR (400 MHz, [D8]THF, 25 °C, TMS): δ = 1.02~1.12(s, 3J(H,H) = 2.4 Hz, 2 H; CH2), 0.9 ppm (t, 3J(H,H) = 8 Hz, 3 H; CH3), 2.83-2.92 ppm (d, 3J(H,H) = 5.7 Hz, 3 H; CH3), 1.05-1.26 ppm (t, 3J(H,H) = 8.7 Hz, 2 H; CH2). These characteristic peak signals confirm that TNYL conjugated TNYL-CSO-SA micelles were successfully synthesized.

Supplementary Figure S2


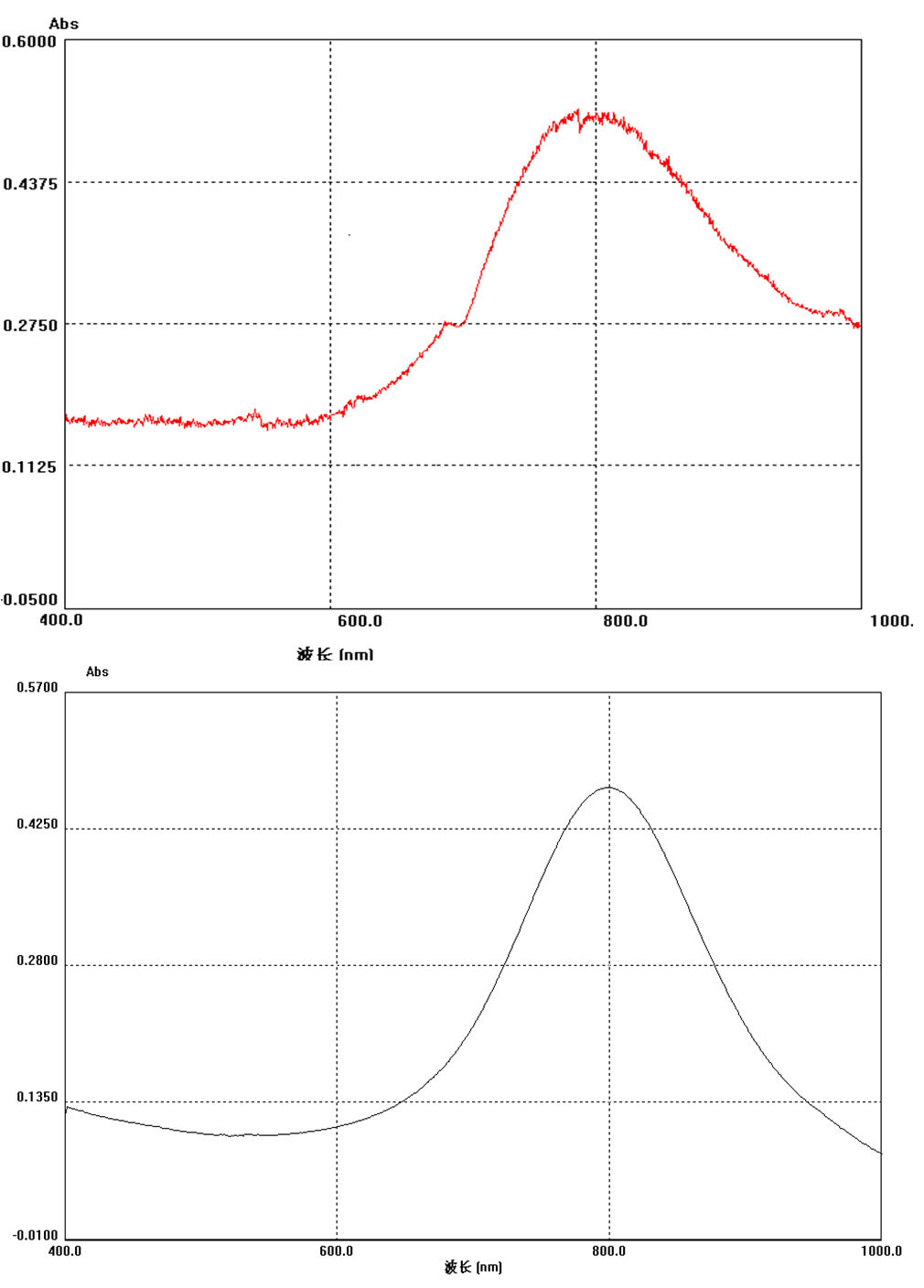


**Supplementary Figure S2.** The ultraviolet absorption spectra of HP-TCS, were recorded on a Beckman Coulter DU-800 UV-visible spectrometer.

Supplementary Figure S3


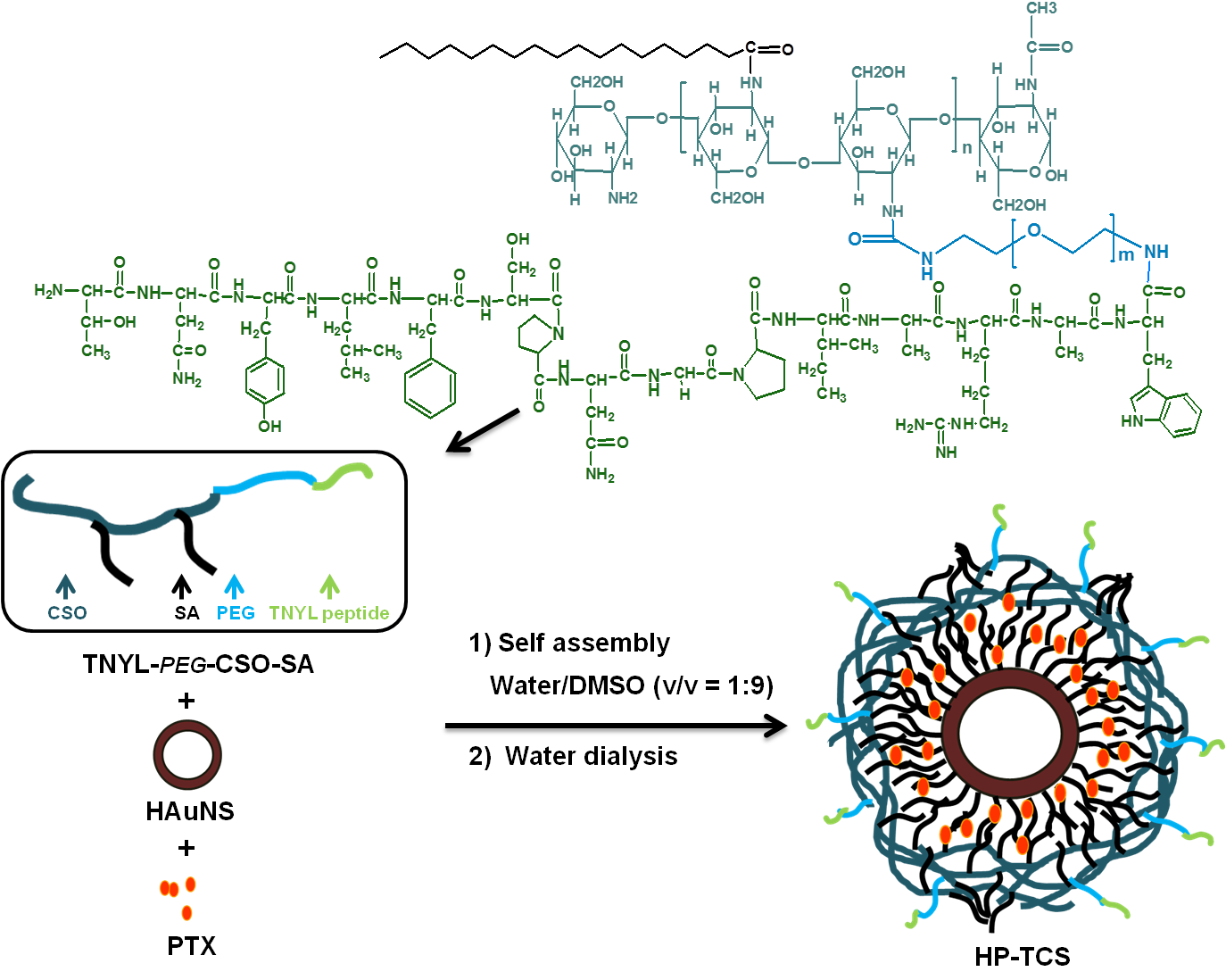


**Supplementary Figure S3.** The schematic illustration of HP-TCS nanoparticles preparation.

Supplementary Figure S4


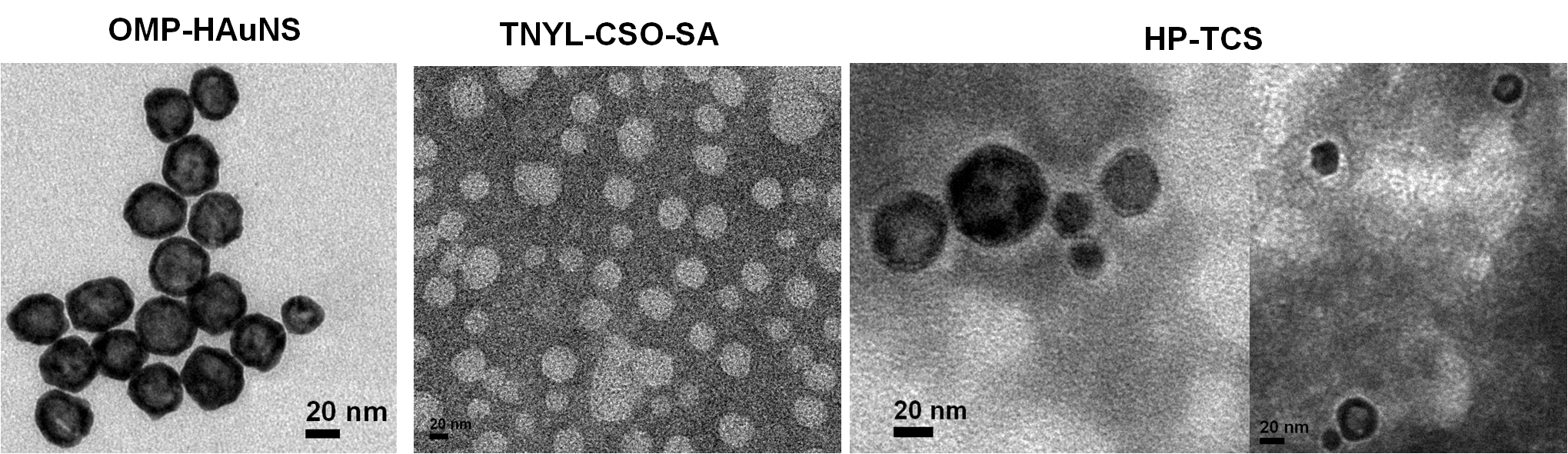


**Supplementary Figure S4.** TEM images with a larger field of OMP-modified HAuNS (a, OMP-HAuNS), TNYL-CSO-SA (b, TCS) micelles and HAuNS & PTX loaded TNYL-CSO-SA (c, HP-TCS) nanoparticles. The sample was stained by phosphotungstic acid (2% W/V) for 40 seconds before the observation. The white spheres indicated the polymer micelles. For HP-TCS nanoparticles, HAuNS was encapsulated in the polymer micelles. More TEM images with large field

Supplementary Figure S5


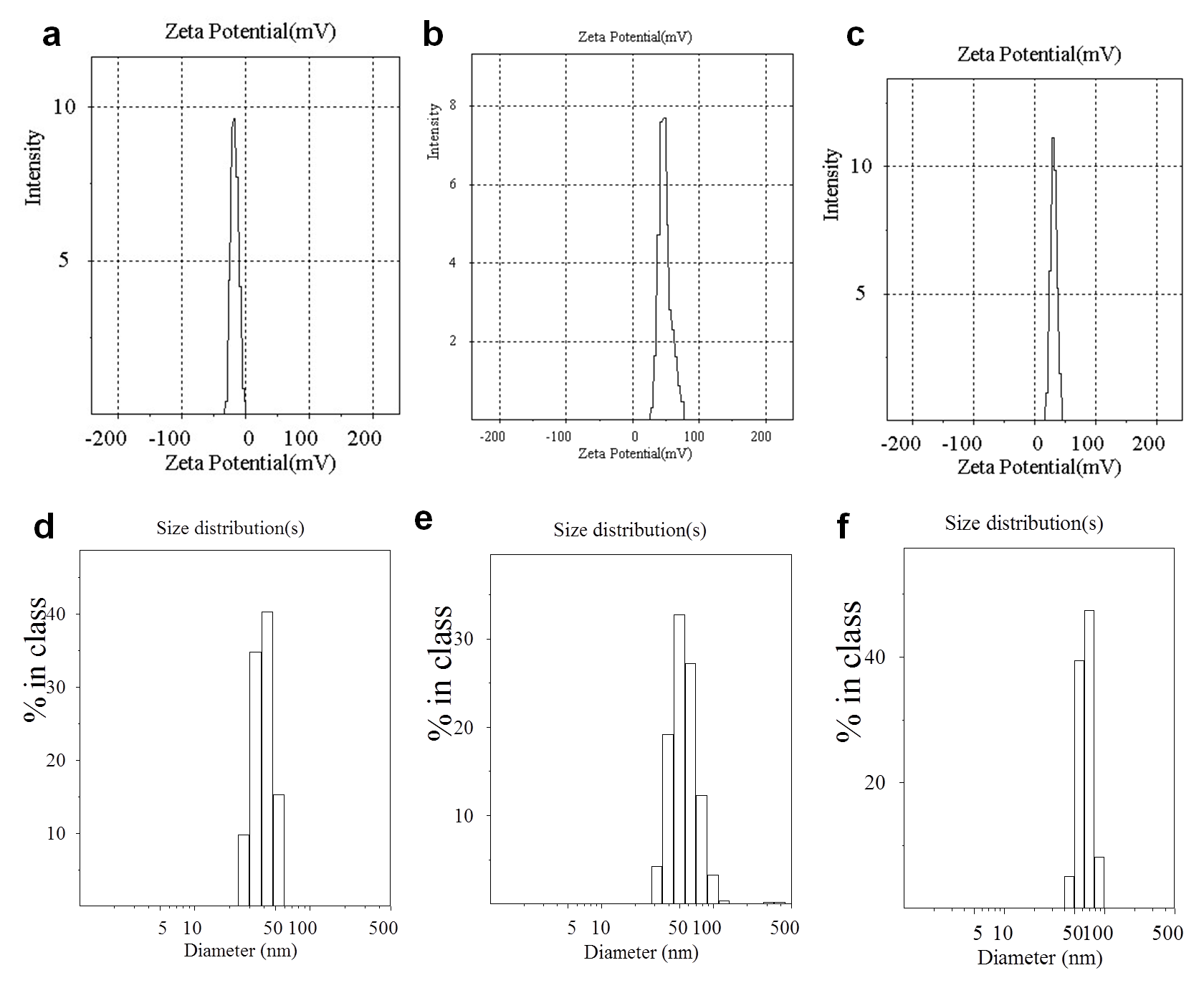


**Supplementary Figure S5.** Size and surface potential of HAuNS (a and d), TNYL-CSO-SA (b and e) and HP-TCS (c and f), measured by a Zetasizer (3000HS, Malvern Instruments Ltd.).

Supplementary Figure S6


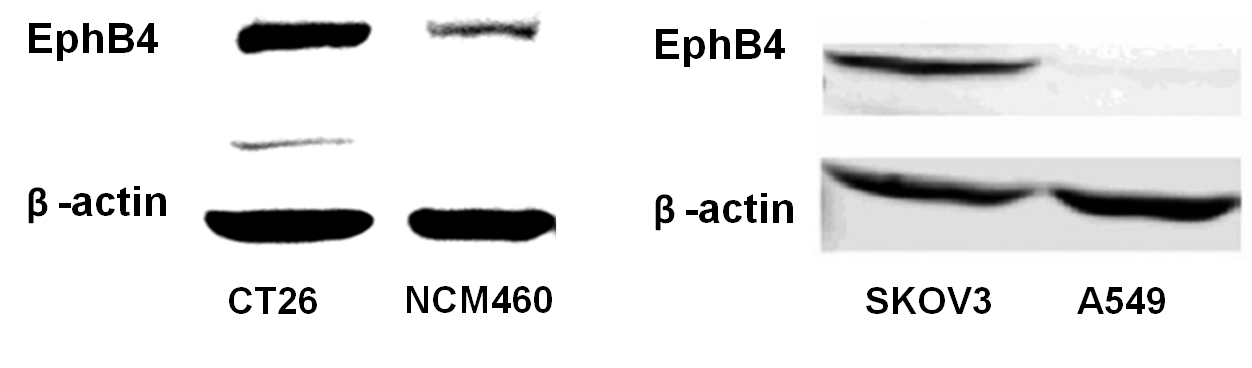


**Supplementary Figure S6.** Western blotting analysis of EphB4 expression in CT26, NCM460, SKOV3 and A549 cell lines.

Supplementary Figure S7

**
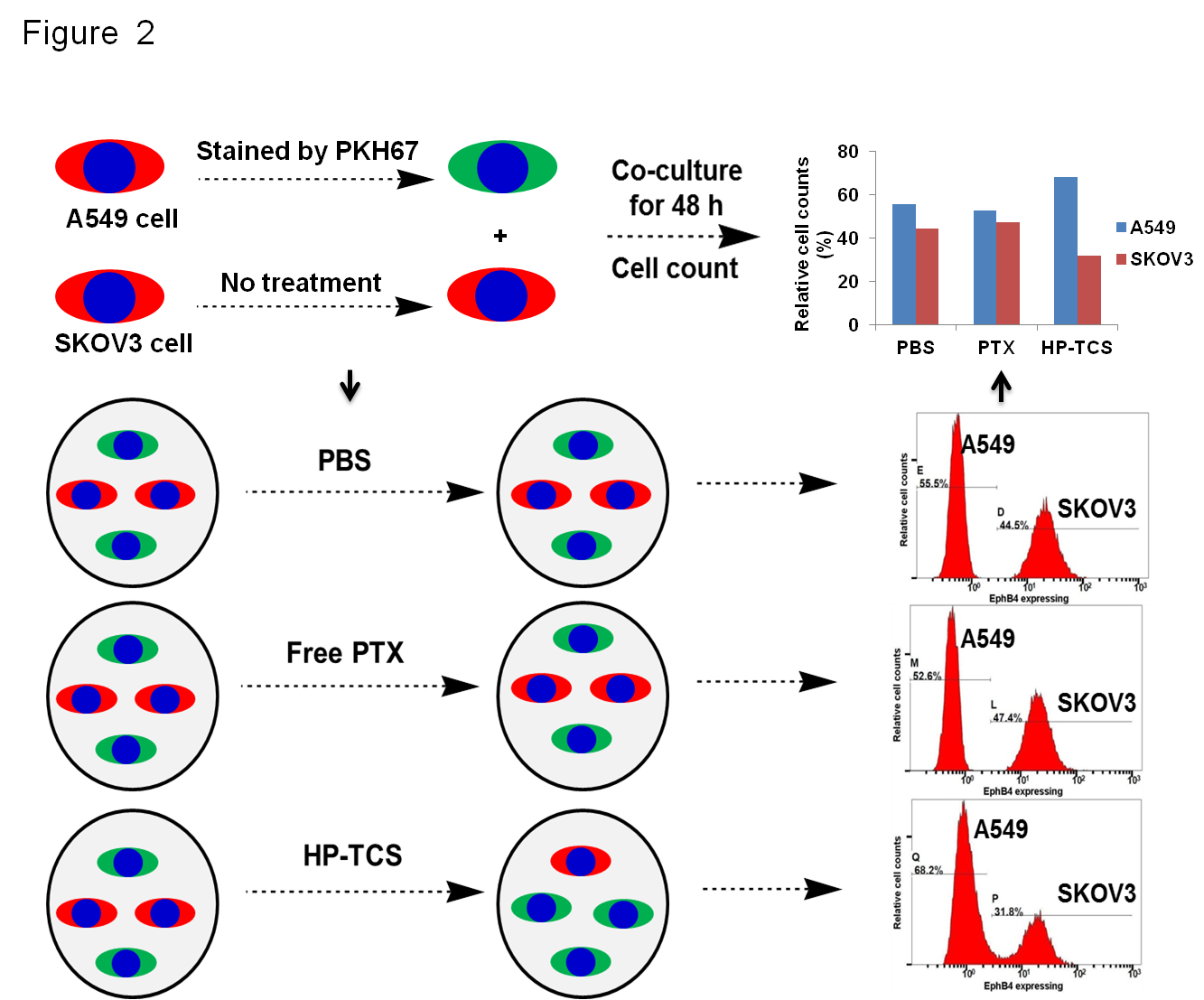
**

**Figures S7.** Specific cytotoxicity test in SKOV3 and A549 cell co-cultured systems in vitro. SKOV3/A549 cells were incubated with no treatment, free PTX and HP-TCS nanoparticles for 48 h and analyzed using a flow-cytometer.

Supplementary Figure S8


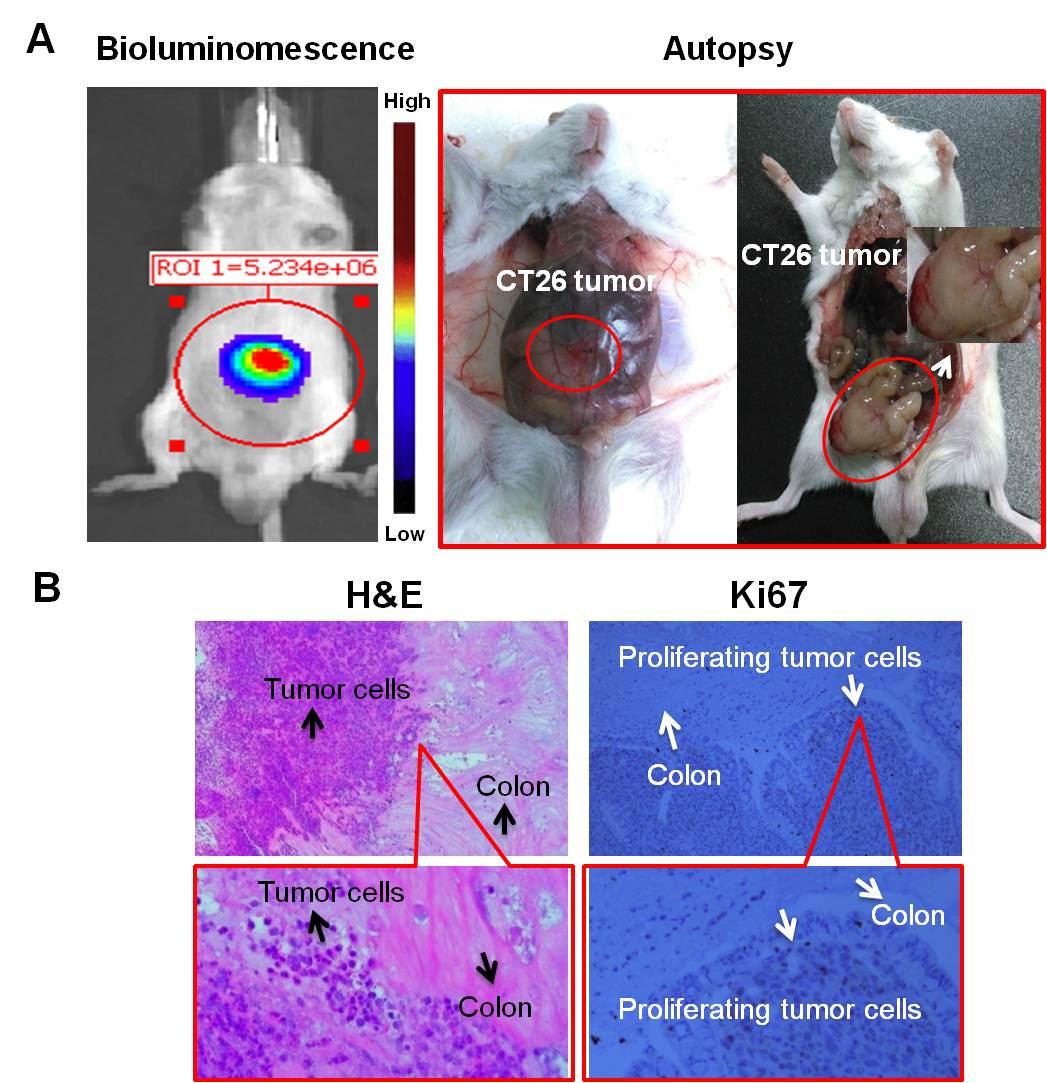


**Supplementary Figures S8.** (A) The *in vivo* bioluminomescence imaging and autopsy photograph of BABL/C mice bearing orthotopic CT26-Luc tumors. (B) Histologic evaluation of CT26-tumor&colon tissues in BABL/C mice bearing orthotopic CT26-Luc tumors, stained with H&E and Ki67.

Supplementary Figure S9

**
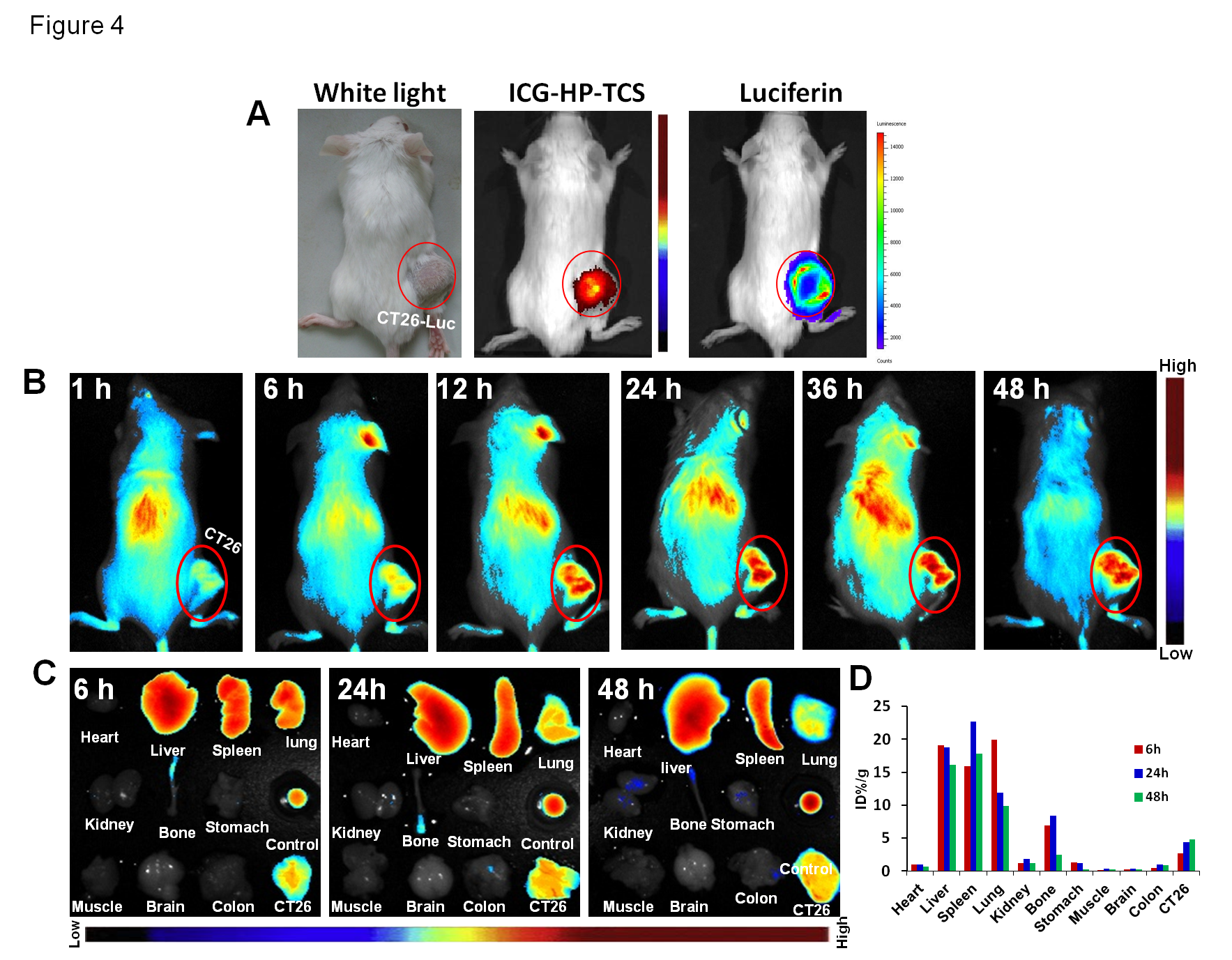
**

**Supplementary Figure S9.** Bioimaging studies in subcutaneous models. (A) The photograph and in vivo imaging of ICG-HP-TCS in CT26-Luc tumor-bearing BABL/C mice after 24 h and at 5 min after ip injection of D-luciferin. (B) The in vivo imaging of the CT26 tumor-bearing BABL/C mice at different times after the iv injection of ICG-labeled HP-TCS nanoparticles. (C) The fluorescent imaging of various tissues at 6 h, 24 h and 48 h after the iv injection of the nanoparticles. (D) The accumulation of HP-TCS nanoparticles in various tissues was calculated as %ID/g (the percentage of the injected dose per gram of tissue). The fluorescent intensity, which indicates the amount of micelles, was read by the imaging system.

Supplementary Figure S10


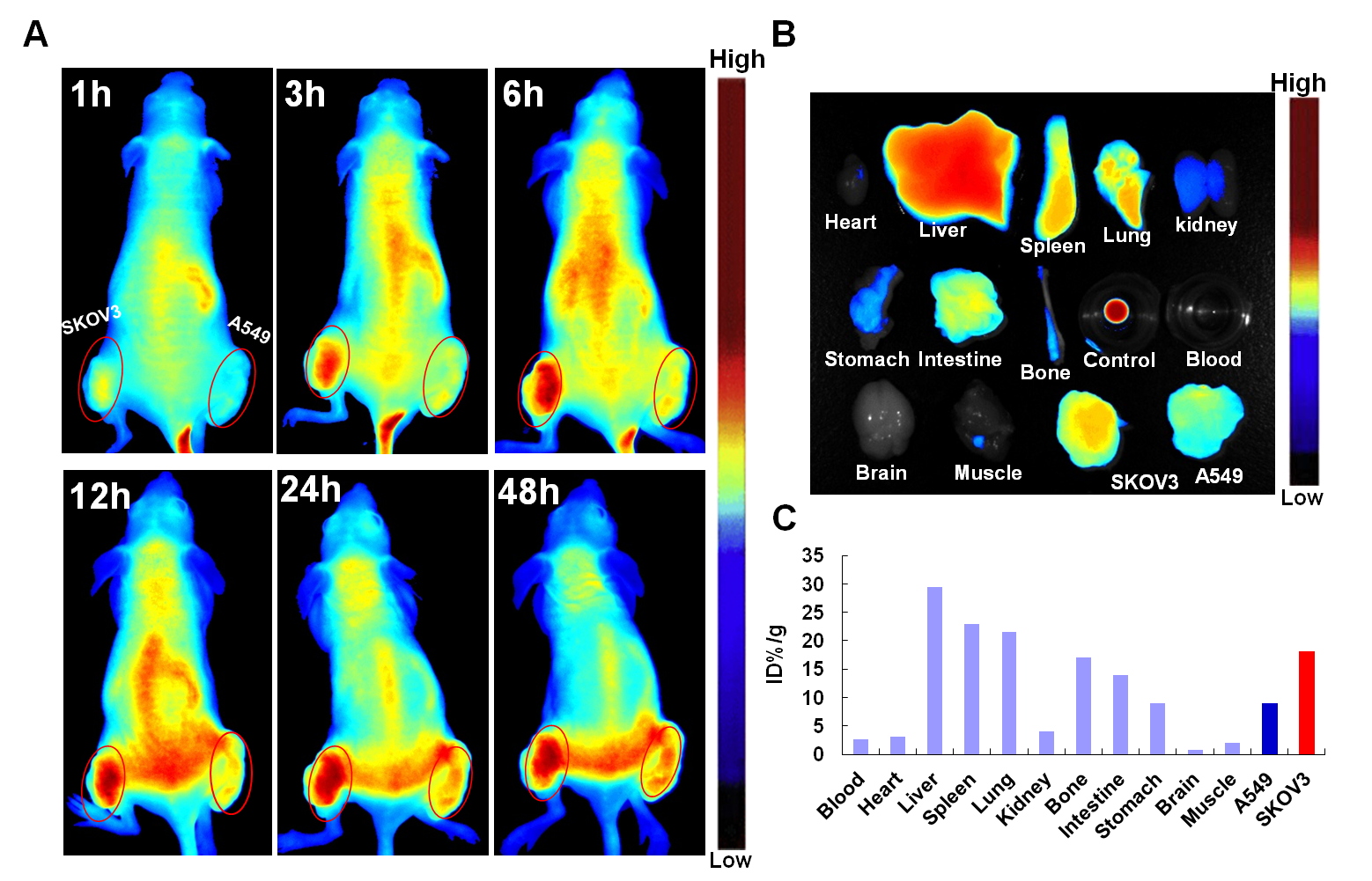


**Supplementary Figure S10.** (A) The *in vivo* imaging of the nude mice, bearing SKOV3 and A549 tumors in left and right side respectively for 48h, at different time after *iv* injection of HP-TCS nanoparticles encapsulating DIR. (B) The ﬂuorescent imaging of various tissues at 48 h after the *iv* injection of the nanoparticles. (C) The accumulation of HP-TCS micelles in various tissues was calculated as %ID/g (the percentage of the injected dose per gram of tissue). The ﬂuorescent intensity, responding the amount of the micelles, was read by the imaging system.

.

Supplementary Figure S11


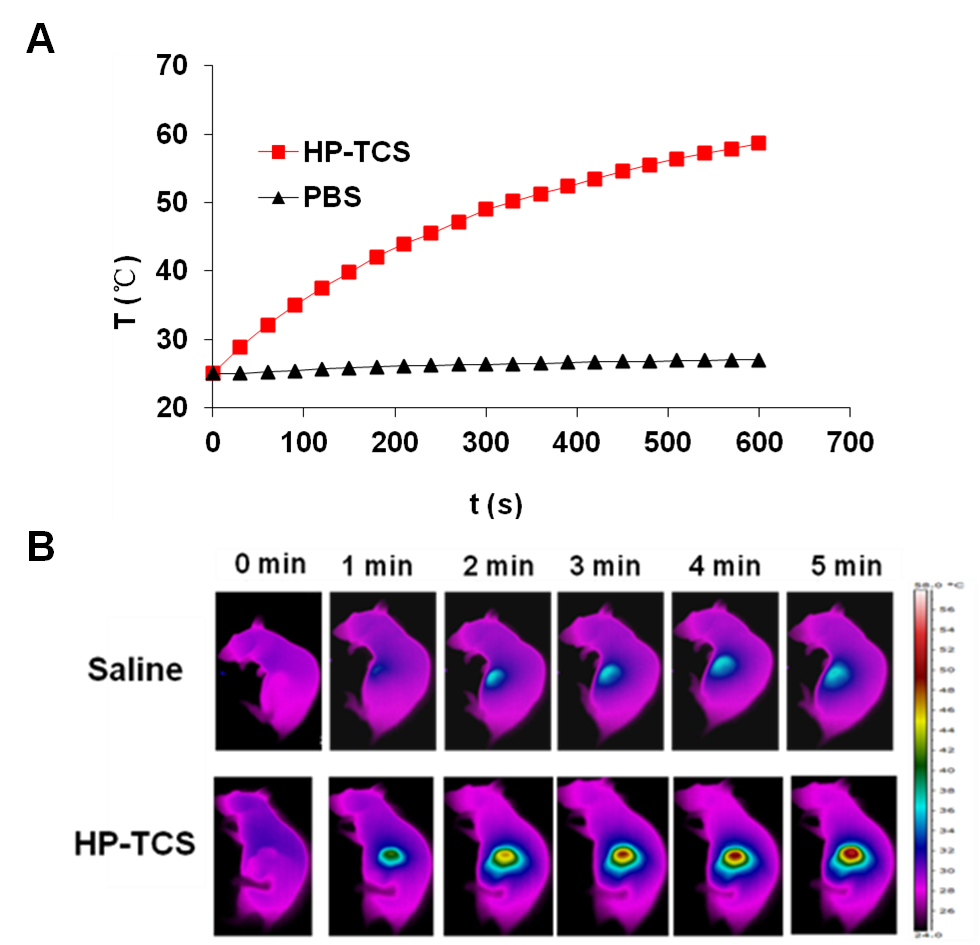


**Supplementary Figure S11.** (A) The temperature changes in aqueous solutions containing HP-TCS nanoparticles (0.5 mg/mL) after exposure to NIR light at an output power of 1.5 W. PBS = phosphate buffered saline. (B) Photothermal images of SKOV3 tumor-bearing nude mice with HP-TCS injection exposed to the NIR laser at power densities of 1W recorded at different time points (0-5 min). IR thermal images of mice with saline injection exposed to the NIR laser were obtained as the control. The scale bar represents the temperature in the range of 24-58 °C.

Supplementary Figure S12


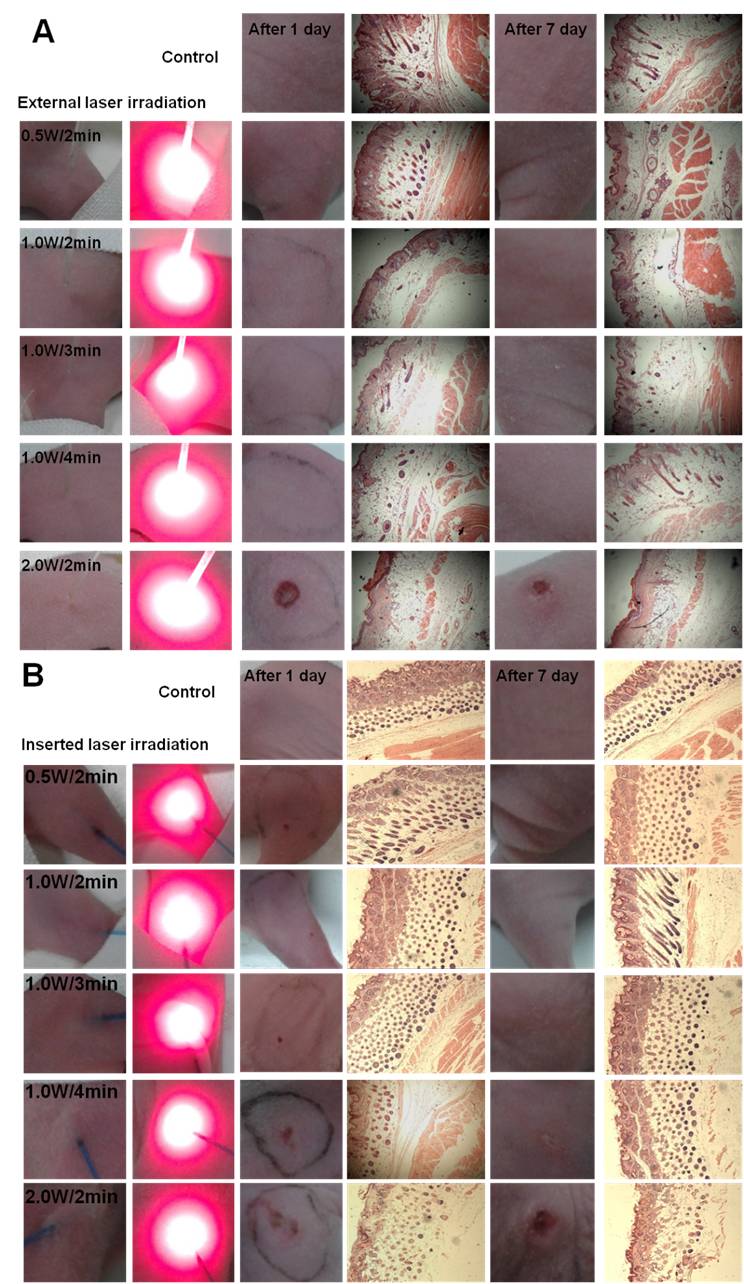


**Supplementary Figure S12.** Histologic evaluation of the normal nude mice on day 1 and after NIR laser treatment. (A) External skin irradiation groups. (B) Inserted skin irradiation groups. Mice were irradiated with the NIR laser light at an output power of 0.5 W/2 min, 1 W/2 min, 1 W/3 min, 1 W/4 min or 2 W/2 min. The mice without NIR irradiation were as control. The skin tissues were stained with H&E.

Supplementary Figure S13


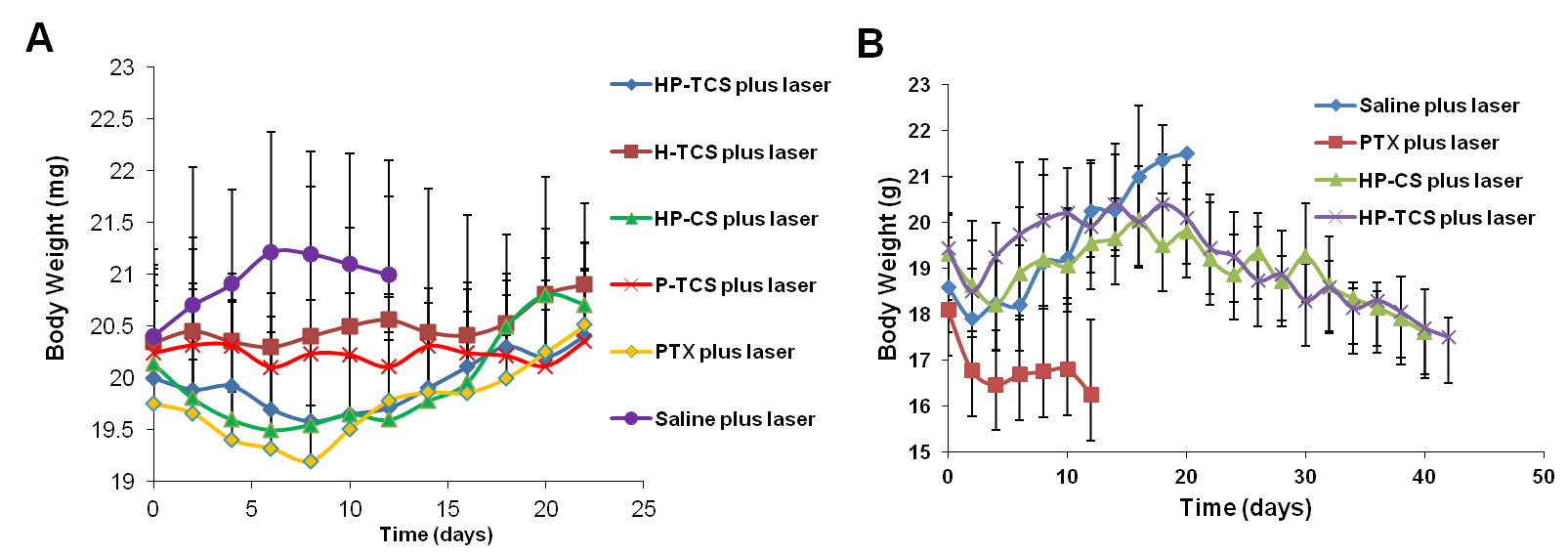


**Supplementary Figure S13.** (A) The body weight changes of the nude mice bearing SKOV3 tumors after *iv* injection of saline, PTX, H-TCS, P-TCS, HP-CS and HP-TCS nanoparticles within 22 days. (B) The body weight changes of the BABL/C mice bearing CT26 tumors after *iv* injection of Saline, PTX, HP-CS and HP-TCS nanoparticles within 22 days. All data represent the mean ± standard deviation (n=6).

Supplementary Figure S14


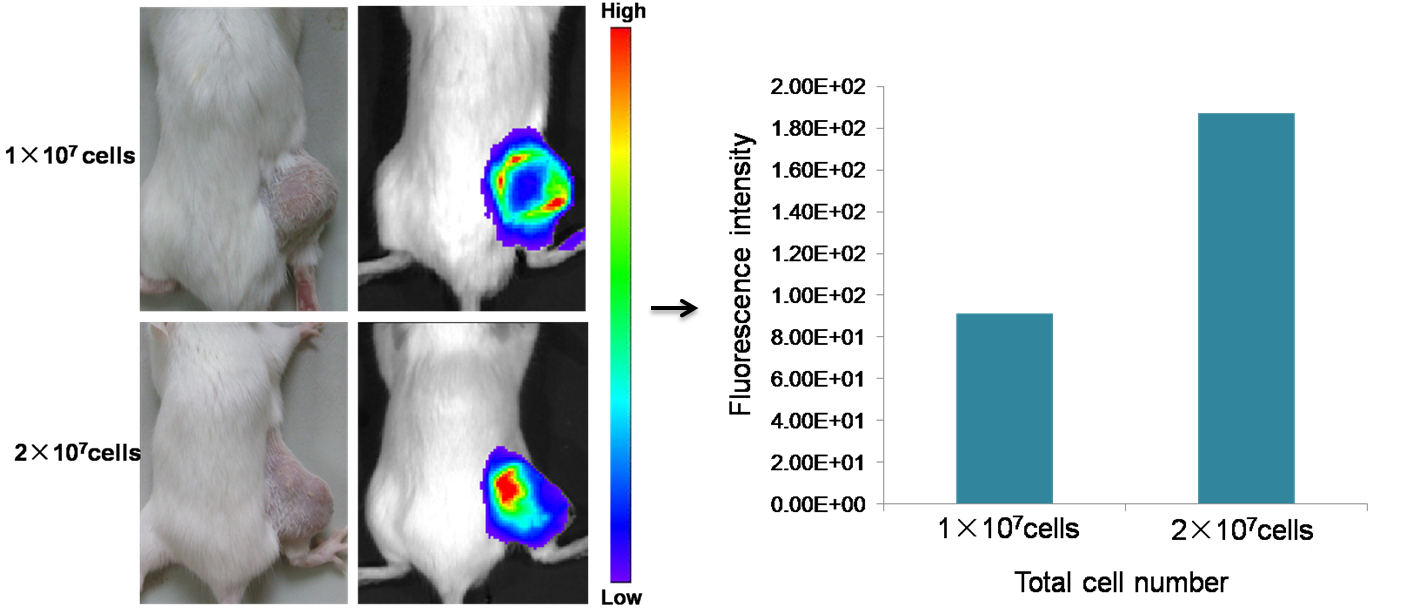


**Supplementary Figure S14.** The *in vivo* imaging and relevant fluorescence intensity of BABL/C mice bearing orthotopic CT26-Luc tumors, injected subcutaneously with different cell numbers in right side, at 5 min after *ip* injection of D-luciferin.

Supplementary Figure S15


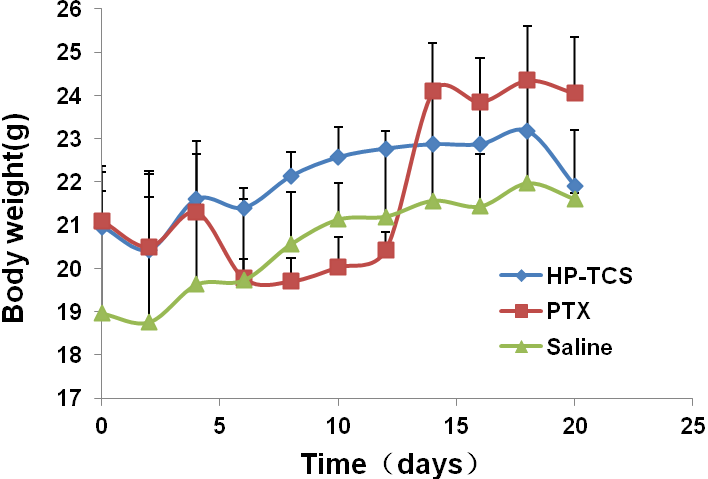


**Supplementary Figure S15.** The body weight changes of BABL/C mice bearing orthotopic CT26-Luc tumors within 21 days. Data represent the mean ± standard deviation (n=3).

Supplementary Table S1

**Table S1.** Size, zeta potential, encapsulated efficiency (EE) and drug content (DC) (n=3)


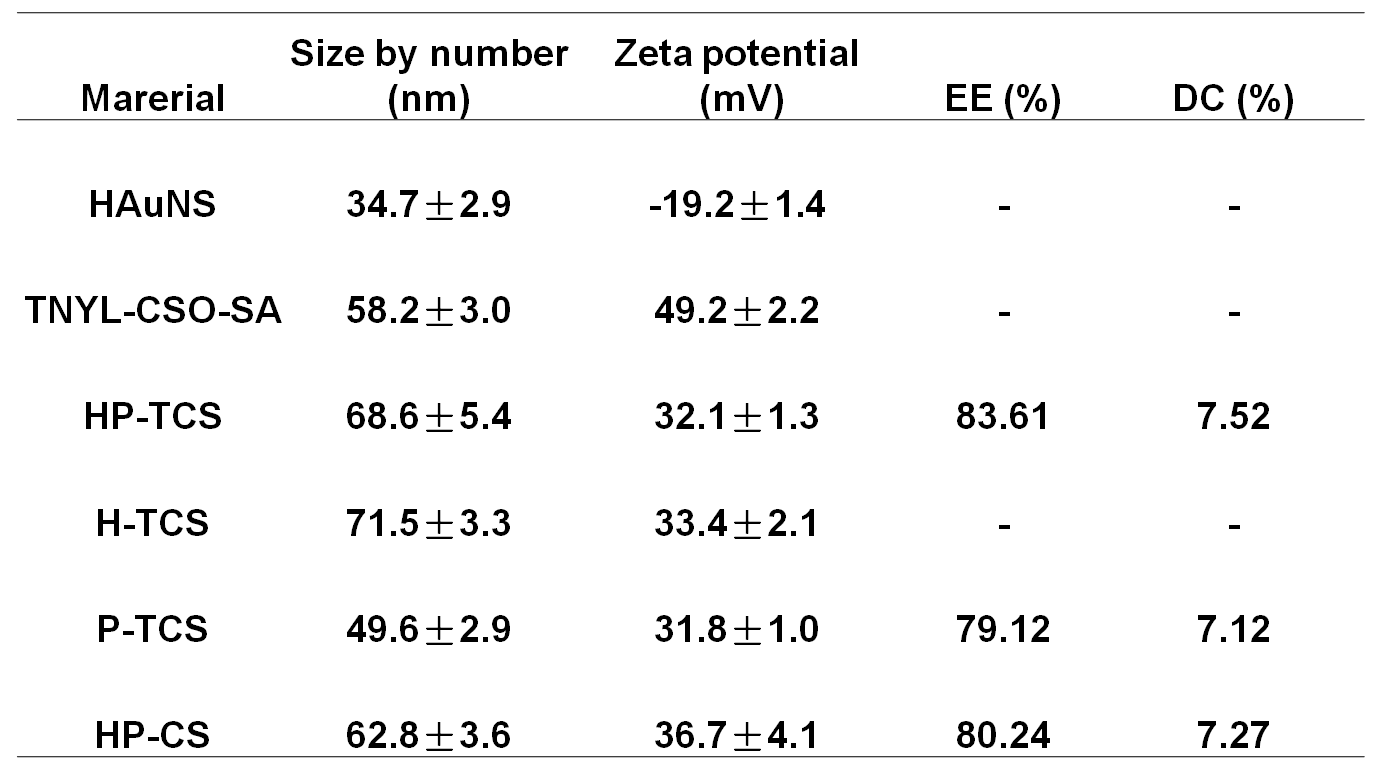


Supplementary Methods 1-4.

**Cell Culture**

A549 (human lung adenocarcinoma) and SKOV3 (human ovarian carcinoma) cells were obtained from Institute of Biochemistry and Cell Biology (Shanghai, China). CT26 (mouse colon adenocarcinoma) and NCM460 (normal mouse colonic epithelia) cells were provided by the First Afﬁliated Hospital of Zhejiang University (Hangzhou, China). CT26-Luc (Luciferase-expressing mouse colon adenocarcinoma) cells were purchased from the Company Limited of Sciencelight Biology Science&Technology (Shanghai, China). A549 and SKOV3 cells were maintained in Roswell Park Memorial Institute (RPMI) 1640 medium containing 10% calf serum (Life Technologies, Inc., Carlsbad, CA) at 37ºC in a humidified atmosphere containing 5% CO_2_. CT26 and CT26-Luc cells were maintained in RPMI 1640 medium (Life Technologies, Inc., Carlsbad, CA) containing 10% fetal bovine serum (Qualified Australian sourced, Gibco) at 37ºC in a humidified atmosphere containing 5% CO_2_. NCM460 cells were maintained in Dulbecco’s modified Eagle’s medium containing 10% fetal bovine serum (Life Technologies, Inc., Carlsbad, CA) at 37ºC in a humidified atmosphere containing 5% CO_2_.

**Synthesis and Hydrophobic Modification of HAuNS**

HAuNS were synthesized using a previously reported method (You J et al., 2010). Briefly, cobalt nanoparticles were first synthesized by deoxygenating deionized water containing 4.5 mL of sodium borohydride (1 M), 2.8 mL of sodium citrate (0.1 M), and 1.0 mL of cobalt chloride (0.4 M). Then, chloroauric acid was added into the solution containing cobalt nanoparticles. The cobalt immediately reduced the gold ions onto the surface of the nanoparticles and was simultaneously oxidized to cobalt oxide. Any remaining cobalt was further oxidized by air, resulting in the final product, HAuNS. The size of the HAuNS was determined using dynamic light scattering on a Zetasizer (3000 HS; Malvern Instruments, UK).

HAuNS was further modified hydrophobically by octadecyl-3-Mercaptopionate (OMP). Briefly, 0.2 mL of HAuNS (200 optical density (OD), 10.0 mg/mL) was centrifuged at 10,000 rpm for 10 min. Then, the obtained pellets were re-dispersed in 1 mL of DMF solution, and 0.1 mmol OMP was added to the DMF solution containing HAuNS. After overnight stirring at room temperature, OM-modified HAuNS (OMP-HAuNS) was purified by centrifugation (10,000 rpm for 10 min), followed by washing twice with DMF. The product was identified by FT-IR analysis. UV-visible spectra were recorded on a DU-800 UV-visible spectrometer (Beckman Coulter, Inc.).

**Synthesis of TNYL Functionalized CSO-SA**

Stearic acid grafted chitosan (CSO-SA) was synthesized as described in our previous report (Hu FQ et al., 2006). Briefly, 2.0 g of CSO was dissolved in 50 mL of distilled water. Then, 1.75 g of SA and EDC (10 mol/mol of SA) were dissolved in 40 mL of ethanol under stirring for 50 min at 60 °C. Then, the CSO solution was heated to 80 °C under vigorous stirring accompanied by the drop wise addition of SA-EDC solution. The reaction lasted 5 h, and the byproducts were removed via ultrafiltration using the Millipore Labscale TFF system (molecular weight cutoff (MWCO) 10 000, Billerica, MA). The obtained product was lyophilized (Labconco, FreeZone 2.5 Plus, Kansas City, MO), further washed with ethanol, and separated by centrifugation (3K30, Sigma Labrorzentrifugen GmbH, Germany).

TNYL, a tumor-targeted peptide, was further conjugated to the surface of CSO-SA micelles. Briefly, 20 mg of TNYL was mixed with (Boc)_2_O (TNYL:(Boc)_2_O = 1:5.2, mol/mol) in dried DMF followed by stirring with light protection at room temperature for 12 h. Then, 23.8 mg of NH_2_-PEG_2000_-NH_2_ and 22.8 mg of EDC were added to the above solution. Succinimidyl t-Boc-TNYL-PEG_2000_-NH_2_ was obtained via a 9-h reaction after the addition of DSC (NH_2_-PEG_2000_-NH_2_:DSC = 1:1, mol/mol) and further reacted with CSO-SA (CSO-SA: succinimidyl t-Boc-TNYL-PEG_2000_-NH_2_ = 1:1, mol/mol) for 24 h. Finally, the protection of (Boc)_2_O to the TNYL peptide was removed by adding hydrochloric acid to the solution. The pH value of the solution was adjusted back to 7.0 using sodium hydroxide. For purification, the solution was dialyzed using a membrane (MWCO 7 kDa, Spectrum Laboratories) against distilled water for 24 h. The final TNYL-conjugated COS-SA (TNTL-CSO-SA) was obtained by lyophilization (Hybaid SNL315SV, UK) and identified by ^1^H-NMR spectroscopy (Bruker AV500, Swiss).

**In Vitro Anti-tumor Activity**

Anti-tumor activity of our micelles *in vitro* was investigated by evaluating their cytotoxicity. Briefly, A549 cells were stained with PKH67 Fluorescent Cell Linker using the above method. PKH67-labeled A549 cells were co-cultured with SKOV3 cells in the same well of a 24-well plate. Then, the mixed cells were separately incubated with PBS (no treatment), free PTX (2 μg/mL) and HP-TCS micelles (2 μg equivalent PTX/mL) for 48 h. The cells were washed thrice with PBS and collected using the trypsin digestion method. The relative survival cell counts between SKOV3 and A54 cells were measured using a flow-cytometer (FC500MCL, Beckman Coulter).
